# T Cell Responses to Human Endogenous Retroviruses in HIV-1 Infection

**DOI:** 10.1371/journal.ppat.0030165

**Published:** 2007-11-09

**Authors:** Keith E Garrison, R. Brad Jones, Duncan A Meiklejohn, Naveed Anwar, Lishomwa C Ndhlovu, Joan M Chapman, Ann L Erickson, Ashish Agrawal, Gerald Spotts, Frederick M Hecht, Seth Rakoff-Nahoum, Jack Lenz, Mario A Ostrowski, Douglas F Nixon

**Affiliations:** 1 Division of Experimental Medicine, Department of Medicine, University of California San Francisco, San Francisco, California, United States of America; 2 Department of Immunology, University of Toronto, Toronto, Ontario, Canada; 3 Gladstone Institute of Virology and Immunology, University of California San Francisco, San Francisco, California, United States of America; 4 Positive Health Program, Department of Medicine, San Francisco General Hospital, University of California San Francisco, San Francisco, California, United States of America; 5 Department of Immunobiology, Yale University School of Medicine, New Haven, Connecticut, United States of America; 6 Department of Molecular Genetics, Albert Einstein College of Medicine, Bronx, New York, United States of America; 7 St. Michael's Hospital, Toronto, Ontario, Canada; University of Pennsylvania School of Medicine, United States of America

## Abstract

Human endogenous retroviruses (HERVs) are remnants of ancient infectious agents that have integrated into the human genome. Under normal circumstances, HERVs are functionally defective or controlled by host factors. In HIV-1-infected individuals, intracellular defense mechanisms are compromised. We hypothesized that HIV-1 infection would remove or alter controls on HERV activity. Expression of HERV could potentially stimulate a T cell response to HERV antigens, and in regions of HIV-1/HERV similarity, these T cells could be cross-reactive. We determined that the levels of HERV production in HIV-1-positive individuals exceed those of HIV-1-negative controls. To investigate the impact of HERV activity on specific immunity, we examined T cell responses to HERV peptides in 29 HIV-1-positive and 13 HIV-1-negative study participants. We report T cell responses to peptides derived from regions of HERV detected by ELISPOT analysis in the HIV-1-positive study participants. We show an inverse correlation between anti-HERV T cell responses and HIV-1 plasma viral load. In HIV-1-positive individuals, we demonstrate that HERV-specific T cells are capable of killing cells presenting their cognate peptide. These data indicate that HIV-1 infection leads to HERV expression and stimulation of a HERV-specific CD8+ T cell response. HERV-specific CD8+ T cells have characteristics consistent with an important role in the response to HIV-1 infection: a phenotype similar to that of T cells responding to an effectively controlled virus (cytomegalovirus), an inverse correlation with HIV-1 plasma viral load, and the ability to lyse cells presenting their target peptide. These characteristics suggest that elicitation of anti-HERV-specific immune responses is a novel approach to immunotherapeutic vaccination. As endogenous retroviral sequences are fixed in the human genome, they provide a stable target, and HERV-specific T cells could recognize a cell infected by any HIV-1 viral variant. HERV-specific immunity is an important new avenue for investigation in HIV-1 pathogenesis and vaccine design.

## Author Summary

The human genome contains a number of remnants or fossils of ancient viral infections referred to as human endogenous retroviruses (HERV). Like fossils, these HERV are considered to be dead or inert in most cases. However, we demonstrate that T cells in the human immune system respond to HERV when a person is infected with the human immunodeficiency virus (HIV). The T cells responding to HERV share characteristics with T cells that effectively control cytomegalovirus, a common chronic viral infection. T cells responding to HERV can also kill target cells carrying HERV protein. For some HIV-positive people, the strength of their response against HERV is related to having a lower HIV viral load. This study has important implications for new directions in HIV vaccine research. One of the key obstacles to creating an effective HIV vaccine is overcoming the ability of some of the viral variants produced when HIV replicates to evade the immune responses that the body mounts to control infections. If T cells that recognize HERV can stably target HIV-infected cells, they could be an important factor in controlling HIV infection.

## Introduction

Human endogenous retroviruses (HERV) are the remnants of ancient infectious agents that successfully entered the germ-line, established a truce with the host, and now make up 8.29% of the human genome [1,2]. Most HERVs are normally quiescent, either because the sequences are truncated and full-length transcription does not occur, or because intracellular defenses such as the APOBEC3 proteins keep activity in check [3]. However, two laboratories have recently reconstructed infectious “hybrids” from one family of endogenous retroviral sequences in the human genome, indicating that significant protein coding capacity and activity potential still exist for these endogenous retroviruses [4,5].

When HIV-1 infects a permissive cell, integration occurs within a genomic context of endogenous retroviruses. In HIV-1-infected cells, the virus initiates numerous changes in the cellular environment to enhance its own expression, which also affect the endogenous retroviruses in the genome. Intracellular defense mechanisms are compromised by proteins like Vif, which works against cellular APOBEC proteins, helping to establish a productive HIV infection [6,7]. Additionally, some HERVs have sequences that are recognized by HIV-1 Rev, providing nuclear export for HERV transcripts in HIV-1-infected cells [8]. RNA derived from HERVs is detectable in the plasma of HIV-1-infected individuals [9,10].

HIV-1-infected cells are normally recognized and killed by cytotoxic CD8+ T cells [11,12]. The proteasome collects and degrades proteins produced within infected cells, then human leukocyte antigen (HLA) class I molecules present peptides on the surface of infected cells [13]. This route of antigen presentation brings a number of antigens, both viral and self, to the surface of infected cells. Following the breaking of tolerance, the recognition of self antigens by CD8+ T cells is possible, especially in the setting of neoplastic transformation [14,15]. This suggests that HLA presentation of HERV antigens on the surface of HIV-1-infected cells could prime a CD8+ T cell response against HERV.

In the present study, we detected evidence of HERV production in HIV-1-positive individuals that significantly exceeded that in HIV-1-negative controls, in agreement with previous studies [9,10]. Our aim was to measure the CD8+ T cell response against HERV. We describe and characterize CD8+ T cell responses against HERV antigens in 29 HIV-1-positive study participants and 13 HIV-1-negative and three hepatitis C positive (HCV+) controls. Finally, we discuss the implications of these responses for the progression of HIV-1 infection.

## Methods

### Study Participants

Individuals were selected from participants in the UCSF OPTIONS cohort study [16]. The study was approved by the local institutional review board (UCSF CHR) and individuals gave written informed consent. Peripheral blood mononuclear cell (PBMC) samples from HIV-1 study participants were obtained from donated buffy coats. PBMCs from HCV+ individuals were obtained from the University of Toronto under their IRB. Studies were performed on cryopreserved PBMCs.

### HERV-K Expression Detection

Plasma samples (1 ml) were centrifuged at 2,000*g* and filtered (0.2 μm) prior to RNA collection to remove remaining cellular contaminants. High speed centrifugation (288,244*g* for 2 h at 4 °C in a Beckman SW41 rotor) was used to pellet particles for RNA isolation with Trizol reagent (Invitrogen). Samples were pre-treated with DNAse to eliminate genomic DNA contamination as a source of amplified HERV sequences. Reverse transcriptase (RT)-PCR was performed with cloned AMV RT (Invitrogen) on samples along with control amplifications without RT enzyme. As a calibration standard, cellular transcript expression of HERV and the housekeeping gene β-actin was measured in cDNA prepared from 2.5 × 10^6^ HIV-negative donor PBMCs. Quantification standards were prepared by serial dilution of the cellular cDNA. Quantitative PCR with primers specific for the transcripts of interest was performed on all samples with the ABI Prism 7900HT Sequence Detection System (Applied Biosystems) using SYBR-Green detection. Primer sequence design and physical data were derived from Primer3 (http://frodo.wi.mit.edu/cgi-bin/primer3/primer3_www.cgi). All amplifications were performed under the following conditions: 94 °C (3 min); 36 cycles of 94 °C (10 s), 63 °C (20 s), 72 °C (25 s); 95 °C (15 s), 60 °C (15 s), 95 °C (15 s). PCR was performed using the ABI PRISM 7900HT Sequence Detection System (Applied Biosystems). Expression levels are presented as percentages relative to PBMC-derived standards and represent the means of triplicate reactions. Gel electrophoresis and melting point analysis of PCR products were used to confirm product purity and amplicon size.

### Peptide Selection

Selection of candidate HERV peptides was based on translated HERV protein sequence data compiled from the NCBI databases, Retrosearch [17], and HERVd [18]. HIV-1 peptides were designed from the sequences of known HIV-1 epitopes listed in the Los Alamos National Laboratory HIV immunology database [19]. Antigenic regions of HERV insertions were assigned an HLA restriction with epitope prediction software [20,21] or based on the HLA restriction of corresponding regions of HIV-1 proteins.

### ELISPOT Assay

ELISPOT analysis was performed as previously described [22]. Equivalent antigen concentrations were used for HIV-1 and HERV peptides. Spot totals for duplicate wells were averaged, and all spot numbers were normalized to numbers of IFN-γ spot-forming units (SFU) per 1 × 10^6^ PBMCs. Spot values from medium control wells were subtracted to determine responses to each peptide.

### Multicolor Cytokine Flow Cytometry

PBMCs from an HIV-1-infected individual were stimulated with or without the peptides HIV VY10, HERV-L IQ10, or a cytomegalovirus (CMV) pool (Becton Dickinson) for 6 h with anti-CD28 and brefeldin A. The cells were stained with fluorophore-conjugated antibodies to CD3, CD4, CD8, CCR7, CD27, CD28, CD45RA, interferon-γ, IL-2, and TNF-α to determine phenotype and function and an amine dye to discriminate between live and dead cells (see also Text S1 and Figure S1). Data were acquired with a LSR-II system (Becton Dickinson). At least 100,000 events were collected and analyzed with FlowJo software (TreeStar). The SPICE software was used to assist in the organization and presentation of multicolor flow data.

### ^51^Cr Release Assays

Cryopreserved PBMCs from two study participants who responded to the HERV-L IQ10 peptide were stimulated for 7 d with peptides or pools of each antigen. Autologous, irradiated, peptide-pulsed feeder cells were used to restimulate for an additional 7 d. Cells were tested for their ability to lyse peptide-pulsed, autologous, Epstein-Barr virus (EBV)-transformed B cell lines by measuring the percentage of specific ^51^Cr release.

### Data Analysis

The Mann-Whitney and Spearman rank tests were performed using GraphPad Prism version 4.00 for Windows (GraphPad Software). *p*-Values less than 0.05 were considered significant for all tests.

## Results

**Figure 1:**
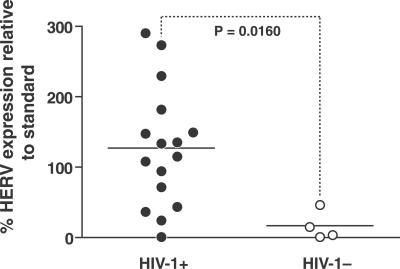
Plasma RNA Levels of HERV-K in HIV-1-Positive and -Negative Individuals' Plasma Levels of a HERV-K transcript derived from the envelope region were measured as a percentage relative to a standard derived from peripheral blood cells. The *p*-value was determined with the Mann-Whitney test.

 We quantified HERV RNA in plasma from 16 untreated HIV-1-positive primary infection individuals and four HIV-1-negative volunteers. We detected significantly greater levels of HERV transcripts in the plasma of most HIV-1-positive individuals compared to controls (Figure 1, Mann-Whitney, *p* = 0.0160).

**Figure 2:**
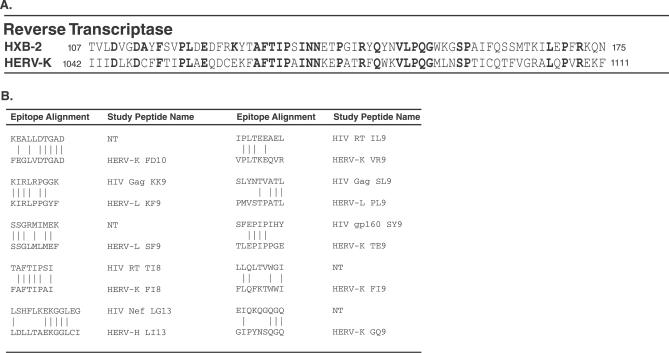
HERV/HIV-1 Amino Acid Alignments Alignments were anchored based on short regions of similarity identified with BLAST [38] short nearly exact match search settings, which included both amino acid similarity estimated with evolutionary matrices (not shown in this figure) and identity. (A) HIV-1 HXB-2 and HERV-K (gi|5802821) showing a segment of the RT protein. Sequence numbering shown is based on position within individual accessions. Identical amino acids are shown in bold. (B) Short regions of similarity between reading frames within different HERV insertions [17,18] in the human genome and HIV-1 HXB-2 and their correspondence to regions of HIV-1. NT is shown when the region in HIV-1 was not a known CD8+ T cell epitope and the HIV-1 peptide was not included in the study.

Although HIV-1 and endogenous retroviruses are phylogenetically distant [23], we identified several regions of clustered and distributed amino acid identity in RT and Gag elements. Sequence comparison within a well-conserved protein like RT showed amino acid identities that were both distributed and concentrated in short, contiguous regions (Figure 2A). Although alignments of the entire amino acid sequence were not possible with less well-conserved proteins, short, contiguous regions of amino acid sequence identity were still present (Figure 2B).

**Figure 3:**
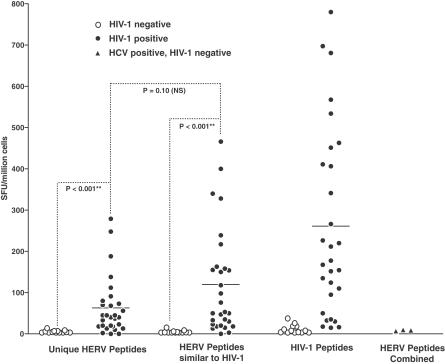
T Cell Responses to HERV and HIV-1 Antigens in HIV-1-Positive and -Negative Individuals Measured by Interferon-γ ELISPOT HERV peptides were grouped according to their similarity to HIV-1 peptide sequence, with “Unique HERV Peptides” having three or fewer amino acids in common with an HIV-1 peptide, and “HERV Peptides Similar to HIV-1” having four or more peptides in common with HIV-1. Subsets of peptides were tested in each patient, with the number tested (*n* = 6–23) varying depending on HLA type. Values shown for responses are normalized per peptide within each grouping (i.e., the sum of the response values to all peptides tested divided by the number of peptides tested for each patient). The individual peptide responses that are summarized in this figure are detailed in Figure S3. Responses in HIV-1-positive individuals are shown as closed circles and in HIV-1-negative individuals as open circles. Responses to all HERV peptides were measured for HCV+ individuals and are shown as filled triangles. *p*-Values are derived from the Mann-Whitney test.

Because of the possibility of both a cross-reactive and independent T cell response to HERV in HIV-1 infection, we sought to measure the CD8+ T cell response to a number of HERV epitopes. We manufactured peptides to test for T cell reactivity, based upon the analysis of HERV sequence with epitope prediction programs [20,21] and based on similarity to known CD8+ T cell epitopes in HIV-1 [19]. A subset of the peptides shared more amino acids in common with HIV-1 (≥4 amino acids), and others were unique to HERV (defined as ≤ 3 amino acids in common) (Figure 2B; Table S1). We tested PBMCs from HIV-1-positive and -negative individuals for HERV- and HIV-1-specific T cell responses in 29 HIV-1-positive study participants from the OPTIONS cohort of primary HIV-1 infection at UCSF [24] and in 13 low-risk HIV-1-negative controls. Specific interferon-γ responses were detected to HERV peptides in HIV-1-infected individuals but not in HIV-1-negative controls (Figure 3, Mann-Whitney, *p* < 0.001). As expected, PBMCs from HIV-1-positive individuals also recognized HIV-1-specific peptides. There was no statistical difference in the mean frequency of responding cells specific for HERV peptides with similarity to HIV-1 sequences and those unique to HERVs (Mann-Whitney, *p* = 0.1025). In addition to HIV-1-negative controls, we also tested three HCV+, HIV-1 negative controls. T cell responses were not detected in response to HERV peptides in HCV+ controls (Figure 3). Individual T cell responses to each peptide tested for each study participant summarized in this figure are shown in detail in Figure S3.

**Figure 4:**
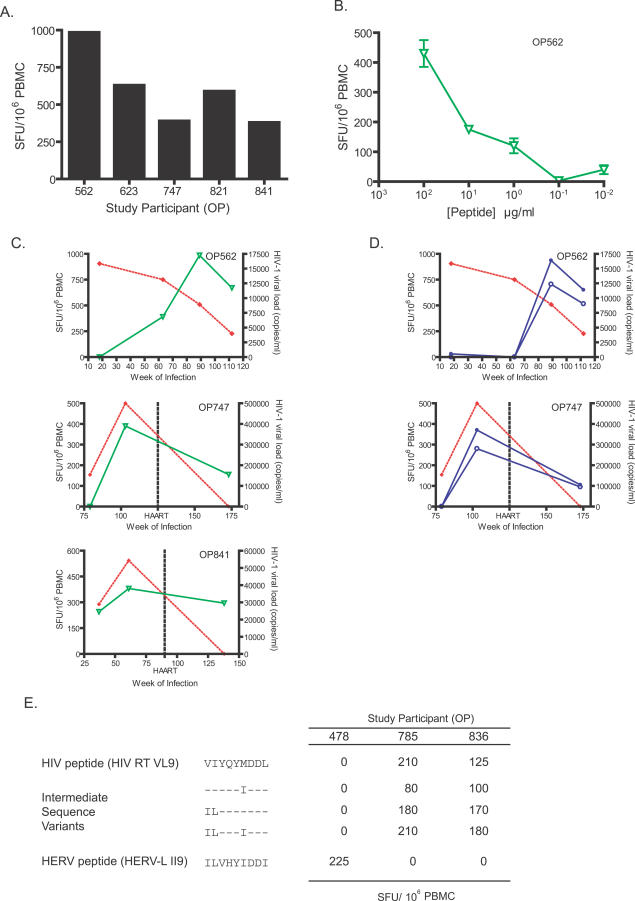
Cross-Sectional and Longitudinal ELISPOT Responses to HIV-1- and HERV-Derived Peptides and Sequence Variants in HIV-1-Positive Study Participants (A) Responses in five HIV-1 positive study participants to the unique HERV peptide HERV-L IQ10. (B) A titration of peptide concentrations for HERV-L IQ10 (open green triangles) peptides for an HIV-1-positive study participant. (C) Responses in three individuals to a unique HERV peptide are shown as inverted green triangles. HIV-1 plasma viral loads are represented as filled red diamonds and are determined with clinical assays for viral load (bDNA and PCR assays). (D) Responses in two representative HIV+ study participants to an HIV-1 and HERV peptide pair with a high level of amino acid identity are shown in blue as open and filled circles, respectively. **(**E) Responses in three study participants to an HLA-A2-restricted HIV-1 epitope peptide (HIV RT VL9) and a HERV-L peptide (HERV-L II9) are shown, along with responses to three intermediate sequence variant peptides in which selected amino acids in the HIV-1 epitope were replaced with the equivalent amino acid found in the HERV peptide.

In a cross-sectional analysis of the cohort of HIV-1-positive study participants, five individuals recognized the unique HERV peptide HERV-L IQ10 with variable magnitudes of response (Figure 4A). In the responder with the highest T cell response magnitude (OP562), a peptide titration assay was performed (Figure 4B).

We measured HERV and HIV-1 T cell responses in three study participants in longitudinal series, including OP562, who naturally contained HIV-1 viremia without antiretroviral therapy over the duration of our longitudinal analysis. The unique HERV peptide HERV-L IQ10 stimulated T cell responses in all three individuals, demonstrating persistent, independent HERV-specific T cell responses at high magnitude (Figure 4C). In two of the individuals tested (OP747 and OP841), highly active antiretroviral therapy (HAART) was initiated, with subsequent declines in HIV-1 plasma viral load and the level of T cell responses to the HERV-L IQ10 peptide.

We also compared responses to HIV-1 and HERV peptides in longitudinal series with a similar pair of peptides. As the peptides HIV Nef LG13 and HERV-H LI13 shared five amino acids, these responses could reflect a level of cross-reactivity. For OP562, responses to both HIV-1 and HERV were not detectable by week 18, but emerged by week 63 of HIV-1 infection (Figure 4D, upper panel). Responses to the HIV Nef LG13 and HERV-H LI13 were detected in another study participant, OP747 (Figure 4D, lower panel).

To address potential cross-reactivity of HERV- and HIV-1-specific T cells in other study participants, we compared responses to an HLA-A2-restricted HIV-1 peptide HIV RT VL9 with responses to a HERV-L peptide, HERV-L II9. The HERV-L II9 peptide is classified as a unique HERV peptide for this study because it shares only three amino acids with its closest corresponding peptide in HIV-1, HIV RT VL9 (see Table 1). To test the effect of amino acid replacements in the HIV-1 peptide that increased the amino acid sequence similarity to the HERV peptide, we included in this analysis a number of intermediate sequence variant peptides, in which selected amino acids in the HIV-1 peptide were replaced with the corresponding amino acid from the HERV sequence (Figure 4E). One individual (OP478) responded to the HERV peptide, but not to the HIV-1 peptide or any of the intermediate sequence variants. Two individuals who responded to the HIV-1 peptide and the sequence variant peptides (to varying degrees) did not respond to the HERV peptide.

**Table 1:**
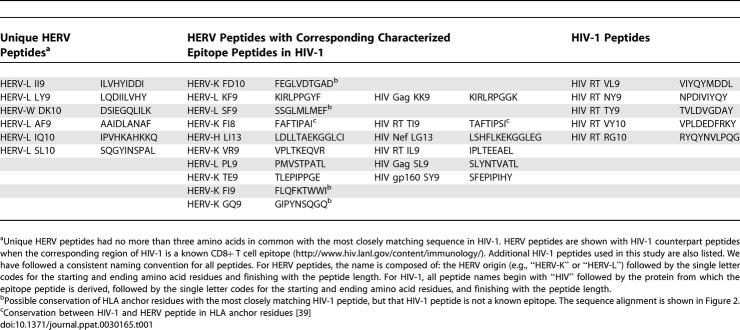
HERV and HIV-1-Derived Peptides Tested in This Study, Divided into the Three Categories Based on Amino Acid Sequence Identity

**Figure 5:**
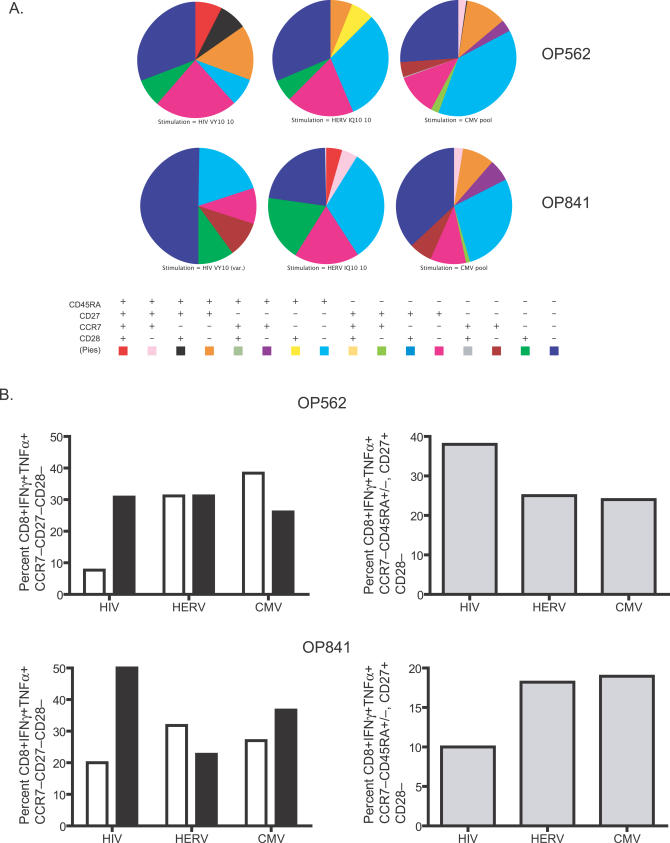
Phenotypic Profile Comparison of HIV-1-, HERV-, and CMV-Specific T Cells Measured by Multicolor Cytokine Flow Cytometry (A) Phenotypic characterization of cytokine producing cells (see Text S1) from two HIV-1-positive study participants for the markers CCR7, CD27, CD28, and CD45RA are shown as pie charts. The colors in the pie charts correspond to the phenotypic categories delineated in the table below them. (B) A subset of the data from (A) is shown, highlighting comparisons of specific phenotypes. The percentages of CD45RA+ (unfilled bars) and CD45RA− (filled bars) T cells are shown in the panels in the left column. The panels in the right column detail the percentages of CD28− T cells from the two study participants.

To qualitatively compare HERV-specific CD8+ T cells with those specific for other viruses, we determined the phenotype and function of HIV-1-, HERV-, and CMV-specific T cells from OP562 and OP841, who responded to the three viruses. For this analysis, we selected HIV-1 and HERV peptides (HERV-L IQ10 and HIV RT VY10) with only two amino acids in common, minimizing potential cross-reactivity. Upon stimulation with respective HERV, HIV-1, and CMV peptides, we ascertained the phenotypes of those cells having a cytokine production profile that were associated with degranulation (Figure 5A; Text S1). The HIV-1-specific T cells of both study participants were skewed towards CD45RA−, whereas CD8+ T cells responding to the HERV peptide had a greater percentage of the terminally differentiated cells (CCR7−CD45RA+) (Figure 5B, left panels). In one study participant (OP562), HERV- and CMV-specific populations shared a lower percentage of CD28−CD27+ CD8+ T cells compared to their HIV-1-specific counterparts (Figure 5B, upper right panel). In contrast, in the other study participant (OP841), HERV- and CMV-specific populations shared a higher percentage of CD28−CD27+ CD8+ T cells (Figure 5B, lower right panel). Overall, the phenotype of the HERV-specific CD8+ T cells more closely resembled the phenotype of CMV-specific than HIV-1-specific T cells.

**Figure 6:**
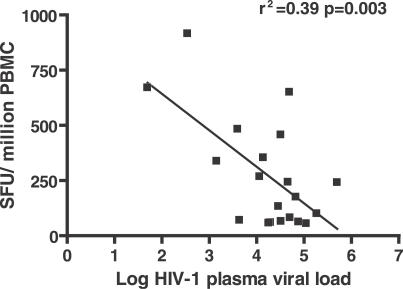
Inverse Correlation between Anti-HERV T Cell Responses and HIV-1 Plasma Viral Load PBMCs from 20 HIV-1-positive individuals not on treatment were analyzed by ELISPOT for HERV responses. The mean response (>50 SFU/million PBMCs) values for all HERV peptides tested had a significant inverse correlation to HIV-1 plasma viral load (Spearman, two-tailed, *r* = −0.49, *p* = 0.03) and by linear regression (*r* ^2^ = 0.39, *p* = 0.003) as shown in the figure.

As these data suggest possible functionality of HERV specific T cells, we measured the relationship of these responses to HIV-1 viral load within the cohort. For the untreated time points available for 20 study participants, HERV-specific T cell responses were significantly inversely correlated with HIV-1 plasma viral load by Spearman non-parametric correlation analysis and linear regression (Spearman, two-tailed, *r* = −0.49, *p* = 0.03; linear regression *r* ^2^ = 0.39, *p* = 0.003; Figure 6).

**Figure 7:**
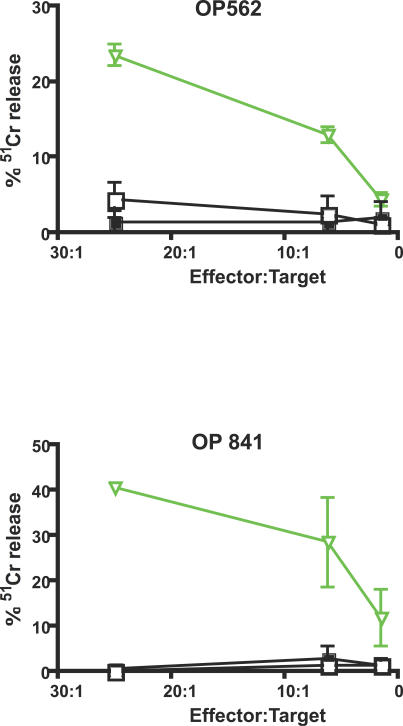
HERV-Specific T Cells Lyse HERV Peptide-Pulsed Targets HERV-L IQ10-specific T cells were tested against autologous B cells pulsed with HERV-L IQ10 peptide (open inverted green triangles), control peptide (filled squares), or no peptide (open squares) in independent peptide-stimulated expansions from two HIV-positive study participants.

Because the ability to control viral load by eliminating infected cells depends on killing, we measured the ability of CD8+ T cells specific for the unique HERV peptide HERV-L IQ10 to kill autologous B cells presenting their target peptide. We peptide stimulated PBMCs from two individuals (OP562 and OP841) to enrich for responsive CD8+ T cells. After a 2-wk peptide stimulation, we used the ^51^Cr-release assay to measure the ability of the enriched CD8+ T cells to kill EBV-transformed B cell targets presenting cognate peptide. CD8+ T cells enriched by stimulation with HERV peptide were able to kill B cell targets presenting their cognate peptide but did not lyse targets loaded with a non-cognate or no peptide (Figure 7). Similar treatment of PBMCs from HIV-1-negative study participants did not produce HERV-specific effectors capable of killing peptide-pulsed targets (Figure S2).

## Discussion

Upon infection of a cell, viruses like HIV-1 alter the cellular environment to favor virus production. Viral proteins act as chaperones to modify controls on RNA trafficking into and out of the nucleus [25,26]. Additionally, cellular suppression mechanisms active against viral transcripts, but not acting at the reverse transcription step, are compromised [6,7,27]. In this altered cellular environment, HERV transcripts could enter translational pathways from which they are normally excluded. While only HERV-K elements contain full-length open reading frames for all proteins, many other HERV insertions in different families retain some form of protein- or peptide-coding capacity relevant for producing a CD8+ T cell response [17,28]. Even HERV elements with premature stop codons that prevent the production of full-length proteins could still produce peptide fragments capable of being presented on HLA molecules, exposing the immune system to HERV antigens.

Our study has identified T cell responses to HERV epitopes in HIV-1-positive individuals. Five participants in the study cohort responded in varying degrees to the unique HERV peptide HERV-L IQ10, and in the subset of those individuals tested longitudinally, those responses persisted over time. This T cell response was titratable over a range of peptide concentrations. Interestingly, the HERV-L IQ10 peptide originates from an open reading frame in the same region (within 31 kbp) of another HERV-L insertion containing a polymorphism associated with improved control of HIV-1 viral load set point [29,30]. While Fellay et al. point out that the effects of the HERV polymorphism on HIV-1 viral load set point are not genetically distinguishable from the effects of HLA-B*5701 in their study, they speculate about a possible antisense mechanism of HIV-1 viral control. The authors also point out that the HERV polymorphism results in an amino acid substitution in one of the predicted encoded proteins. Immunologic mechanisms of control via CD8+ T cell recognition of HERV epitopes are another plausible mechanism for the observed improvement of control over HIV-1 viral load set point. Both full-length, nearly intact viral insertions such as HERV-K, but also older, more heavily disrupted insertions, such as HERV-L, could stimulate important HERV-specific T cell responses.

Since recognition of proteins by T cells occurs by presentation of short peptide fragments, distantly related viruses can still have epitopic regions in common with shared amino acids, even in less well-conserved proteins or when sub-full-length protein transcripts are produced by the cell. Short regions of similarity between HERV and HIV-1 peptide sequences could lead to a level of cross-reactivity for T cell receptors recognizing similar epitopes. Our data demonstrate parallel dynamics of T cell responses for peptides representing regions of similarity between HIV-1 and different HERV. The studies described here established limits to the cross-reactivity by demonstrating T cell responses to different variants of HIV-1 peptide sequences that do not overlap with T cell responses to HERV epitope peptides, but the extent and broader implications of this potential cross reactivity merit further study.

Having identified CD8+ T cell responses to HERV epitopes in HIV-1 infection, it was important to determine the relevancy of these cells in the cellular immune response to HIV-1 infection. One study participant (OP562) was able to control HIV-1 viral load without HAART over the duration of our longitudinal study, and he had the highest observed magnitude of response to a unique HERV peptide. For the entire cohort in a cross-sectional component of the study, the level of T cell responses to HERV was inversely correlated to HIV-1 plasma viral load. Given these data, suggestive of a role for HERV-specific T cells in controlling HIV-1 infection, it was important to compare the phenotype of HERV-specific T cells to those specific for other viruses.

Previous studies of T cells in chronic viral infections of humans have identified differences of phenotype and function between virus-specific CD8+ T cells, which have led to the notion that HIV-1-specific CD8+ T cells are deficient in their maturation, and potentially in function [31,32]. We detected skewing towards CD45RA− for the HIV-1-specific T cells in both study participants analyzed, as has been previously observed for HIV-1 specific CD8+ T cells [32]. CD8+ T cells responding to the HERV peptide had a greater percentage of CCR7−CD45RA+ cells, presumed to be terminally differentiated cells that are associated with improved viral control in HIV-1-specific T cells [33]. Like the HERV-specific CD8+ T cells, CMV-specific CD8+ T cells in our data and in previous studies did not have a phenotypic profile skewed towards CD45RA− [33]. Additionally, HERV-specific CD8+ T cells had a lower percentage of CD28−CD27+ CD8+ T cells compared to their HIV-1-specific counterparts in one study participant, as reported in previous studies [31]. In the other study participant, both HERV- and CMV-specific CD8+ T cells shared a higher percentage of CD28−CD27+ CD8+ T cells compared to their HIV-1-specific counterparts. CD28 expression on the cell surface modulates responsiveness to co-stimulation, and can change according to the activation level of a T cell [34]. Higher levels of CD28 on the T cell surface increase the sensitivity of that T cell to B7 co-stimulation by antigen presenting cells, but engagement of the CD28 receptor leads to its down-regulation in a negative feedback loop [35]. Overall, our data are consistent with previous phenotypic and functional observations for HIV-1-specific CD8+ T cells, and indicate that HERV-specific CD8+ T cells more closely resemble CD8+ T cells induced in controlled chronic viral infections such as CMV.

Controlling HIV-1 viral load also requires killing of infected target cells. HERV antigen production by HIV-1-infected cells would make them a potential target for HERV-specific CD8+ T cells. However, as HERV-specific CD8+ T cells are specific for self-antigens, CD8+ cells responding to HERV peptides may be impaired in their ability to kill targets. Our data show that HERV-specific CD8+ T cells specifically kill B cell targets presenting HERV peptides. Cells capable of killing B cell targets presenting HERV peptides were not detectable in the PBMCs of HIV-1-negative study participants.

The T cell responses against HERV peptides in HIV-1-positive individuals appear to be driven by HIV-1. With effective HIV-1 suppression by HAART, responses to unique HERV peptides and those similar to HIV-1 both undergo a decline. However, chronic viral infections can lead to dysregulation of the CD8+ T cell response [36]. To rule out the effects of chronic viral infection in generating HERV CD8+ T cell responses, we analyzed responses in HCV+ controls. The lack of HERV-specific T cell responses in HCV+ study participants indicates that HERV-specific CD8+ T cell responses are present in HIV-1 but not other chronic viral infections such as HCV.

Our data have important implications for redefining the breadth of epitopes that make up what is considered the “HIV-1-specific” CD8+ T cell response. CD8+ T cell depletion in rhesus macaques has been used to demonstrate the importance of CD8+ T cells in viral control [37]. Of necessity, the depletion was performed regardless of specificity of the CD8+ T cells. Thus, CD8+ T cells with specificities for epitopes other than those derived from the virus itself could have also been depleted in these experiments and be important for viral control. Our results demonstrate HERV-specific T cell responses in individuals with primary HIV-1 infection. While these responses could play a role in HIV-1 pathogenesis by attacking any cell presenting HERV epitopes, it is interesting to consider the potential benefit of these responses to the control of HIV-1. An inverse correlation between anti-HERV T cell responses and HIV-1 plasma viral load demonstrates a role for HERV CD8+ T cell responses in helping to contain HIV-1 viremia. Additionally, the phenotype of HERV-specific CD8+ T cells more closely resembles that of CD8+ T cells generated in the setting of a viral infection with good immunologic control. Our results suggest that HERV-specific CD8+ T cells, both those cross-reactive with HIV-1 epitopes and those specific for their HERV targets, should be included in the repertoire of cells capable of controlling HIV-1 replication. Because one of the greatest challenges for the immune system in effectively and durably controlling retroviral replication is responding to rapidly arising viral variants, it is interesting to speculate on the potential of HERV immunity to aid in broad spectrum and long-term immune control of HIV-1. HERVs are genome-encoded elements with the same sequences present in every cell. An anti-HERV-specific immune response could target any HIV-1-infected cell irrespective of HIV-1 viral sequence. HERV could provide an effective surrogate target for the immune response to eliminate HIV-1-infected cells and bears investigation as candidates for inclusion in a new type of HIV-1 vaccine. In summary, we show HERV-specific immune responses in HIV-1 infection. Manipulation of these responses could play an important role in HIV-1 immunotherapeutics, or augment HIV-1 vaccine strategies.

## Supporting Information

Figure S1 Gating Strategy for Multicolor Cytokine Flow Cytometry (328 KB PDF)

Figure S2 CD8+ T Cell Peptide Expansions from HIV-1-Negative Study Participants (180 KB PDF)

Figure S3 Supplement to Figure 3
Detailed responses to all peptides tested on all individuals summarized in Figure 3 of the text.
(385 KB PDF)

Table S1 Additional Peptide Data Including HERV Subtypes with Accession Numbers and HIV HXB-2 Protein Location and HLA Restrictions (67 KB DOC)

Table S2 Primer Sequences for HERV Amplification (Table S2a) and Sequences Obtained from HERV-K Env Amplification (Table S2b) (33 KB DOC)

Text S1 Supplemental Methods (26 KB DOC)

### Accession Numbers

HERV-L ORF accession numbers from Retrosearch [17] are 162563, 162568, 162604, 162605, and 162613. The National Center for Biotechnology Information (http://www.ncbi.nlm.nih.gov/) accession numbers for the proteins discussed in this paper are HERV-H (gi|44887889); HERV-K (gi|52001472, gi|5802821, gi|75766508, gi|67782351); and HERV-W (gi|52000737).

## Abbreviations

CMV: cytomegalovirus
HAART: highly active antiretroviral therapy
HCV+: hepatitis C positive
HERV: human endogenous retrovirus
HLA: human leukocyte antigen
PBMC: peripheral blood mononuclear cell
RT: reverse transcriptase
