# Supplementary material for: T Cell Responses to Human Endogenous Retroviruses in HIV-1 Infection
Source: PLoS Pathog. 2007 Nov 9;3(11):e165. doi: 10.1371/journal.ppat.0030165 (PMC2065876; doi:10.1371/journal.ppat.0030165)
Supplement: Supplementary file 1 [file ppat.0030165.sg001.pdf]

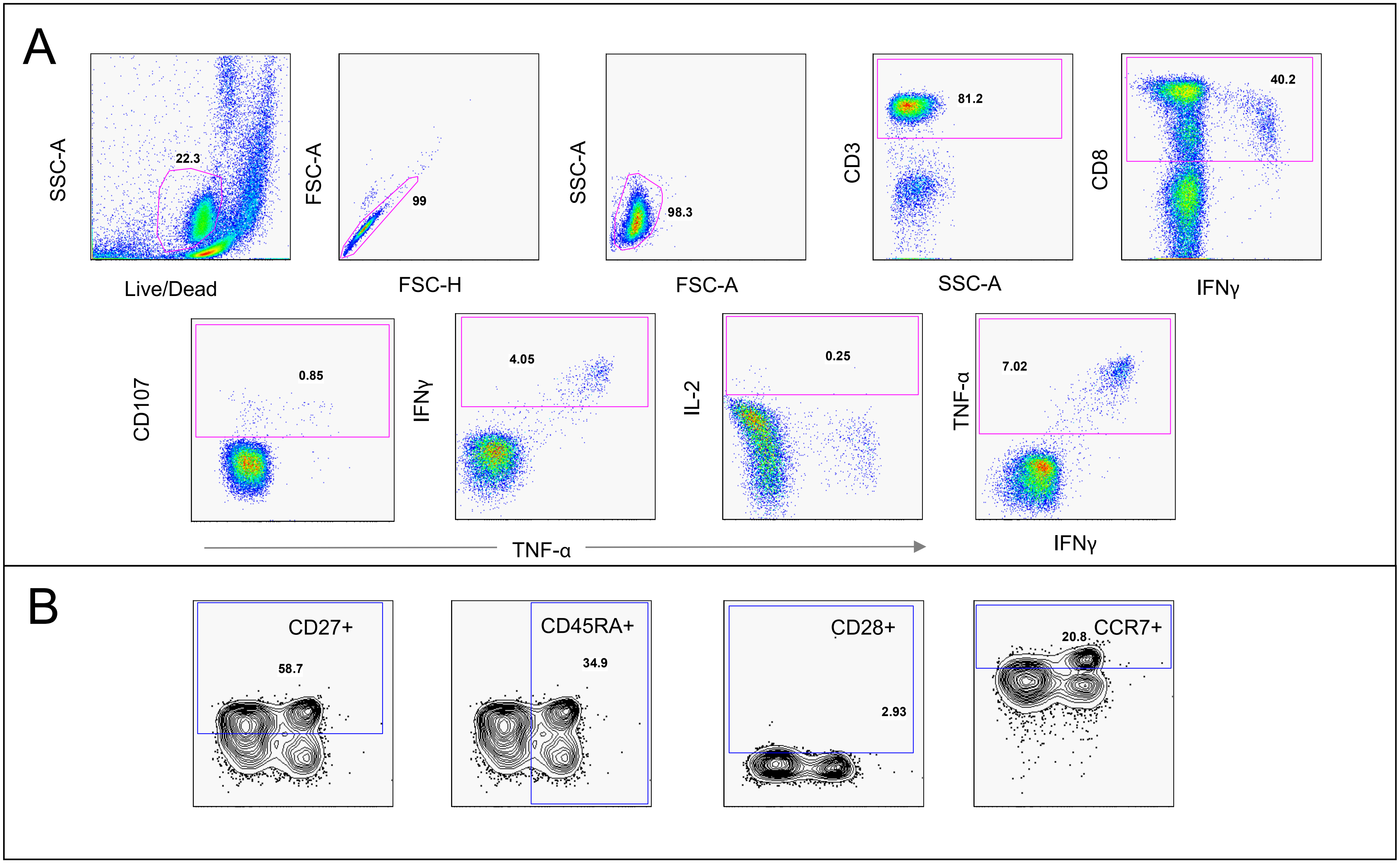
A
SSC-A
22.3
Live/Dead
FSC-A
99
FSC-H
SSC-A
98.3
FSC-A
CD3
81.2
SSC-A
CD8
40.2
IFNγ
CD107
0.85
IFNγ
4.05
IL-2
0.25
TNF-α
7.02
IFNγ
TNF-α
B
CD27+
58.7
CD45RA+
34.9
CD28+
2.93
CCR7+
20.8
