# Supplementary material for: T Cell Responses to Human Endogenous Retroviruses in HIV-1 Infection
Source: PLoS Pathog. 2007 Nov 9;3(11):e165. doi: 10.1371/journal.ppat.0030165 (PMC2065876; doi:10.1371/journal.ppat.0030165)
Supplement: Supplementary file 2 [file ppat.0030165.sg002.pdf]

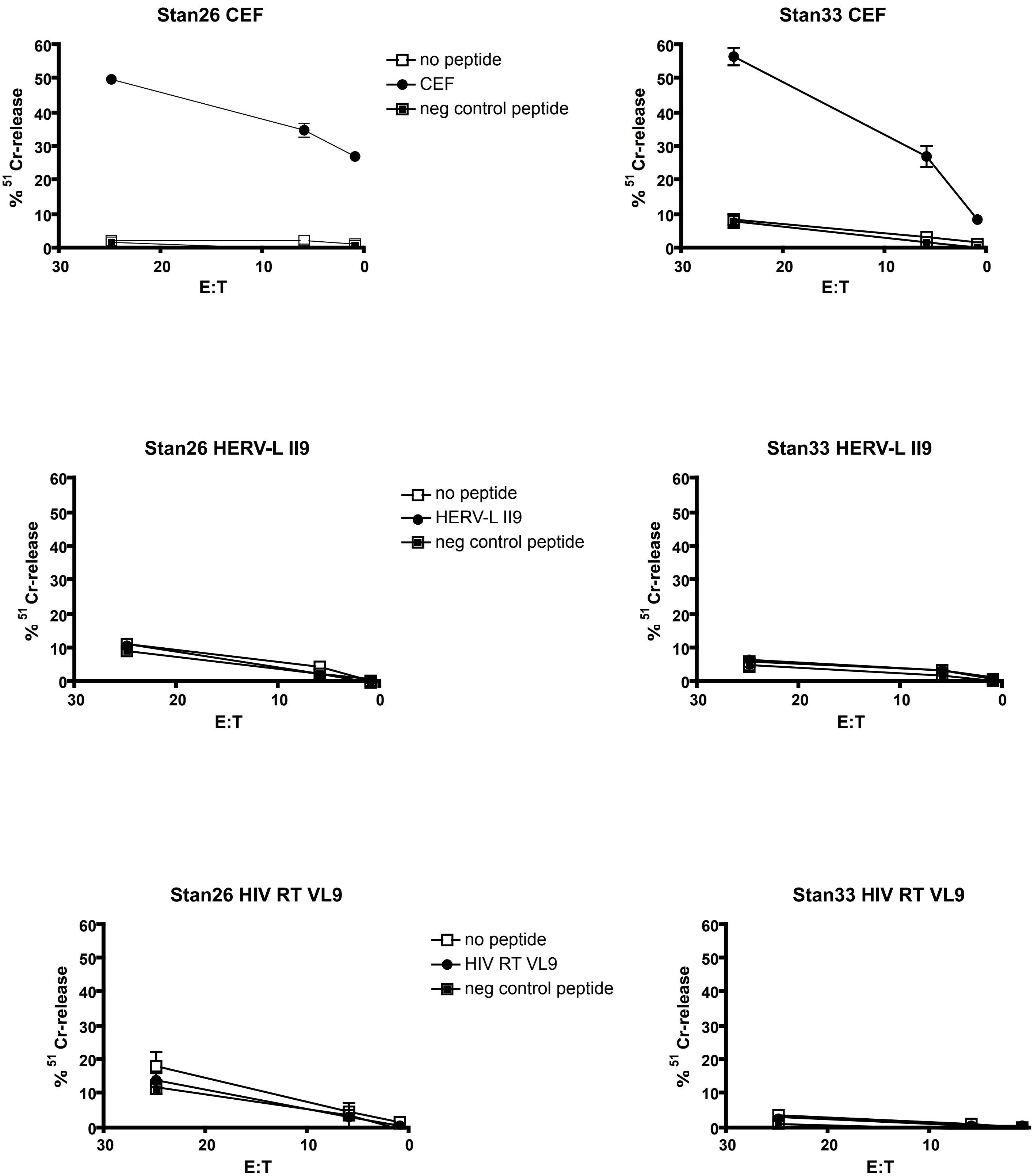

Stan26 CEF
Stan33 CEF
no peptide
CEF
neg control peptide
Stan26 HERV-L II9
Stan33 HERV-L II9
no peptide
HERV-L II9
neg control peptide
Stan26 HIV RT VL9
Stan33 HIV RT VL9
no peptide
HIV RT VL9
neg control peptide
% 51 Cr-release
E:T
