# Supplementary material for: T Cell Responses to Human Endogenous Retroviruses in HIV-1 Infection
Source: PLoS Pathog. 2007 Nov 9;3(11):e165. doi: 10.1371/journal.ppat.0030165 (PMC2065876; doi:10.1371/journal.ppat.0030165)
Supplement: Supplementary file 3 [file ppat.0030165.sg003.pdf]

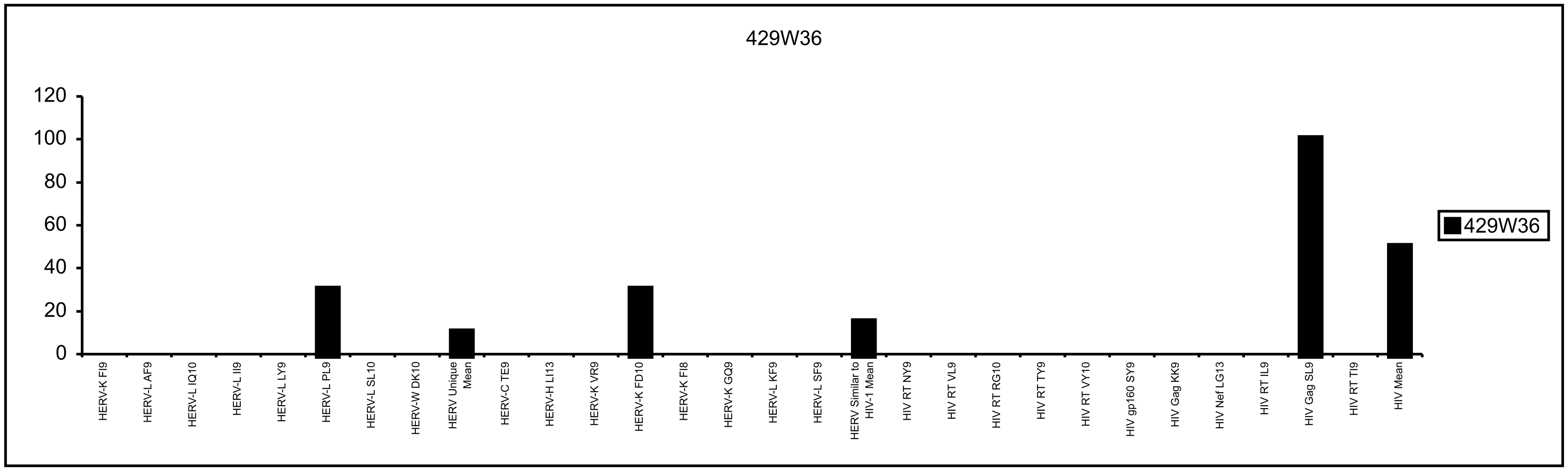
429W36
120
100
80
60
40
20
0
429W36
HERV-K FI9
HERV-L AF9
HERV-L IQ10
HERV-L II9
HERV-L LY9
HERV-L PL9
HERV-L SL10
HERV-W DK10
HERV Unique Mean
HERV-C TE9
HERV-H LI13
HERV-K VR9
HERV-K FD10
HERV-K FI8
HERV-K GQ9
HERV-L KF9
HERV-L SF9
HERV Similar to HIV-1 Mean
HIV RT NY9
HIV RT VL9
HIV RT RG10
HIV RT TY9
HIV RT VY10
HIV gp160 SY9
HIV Gag KK9
HIV Nef LG13
HIV RT IL9
HIV Gag SL9
HIV RT TI9
HIV Mean

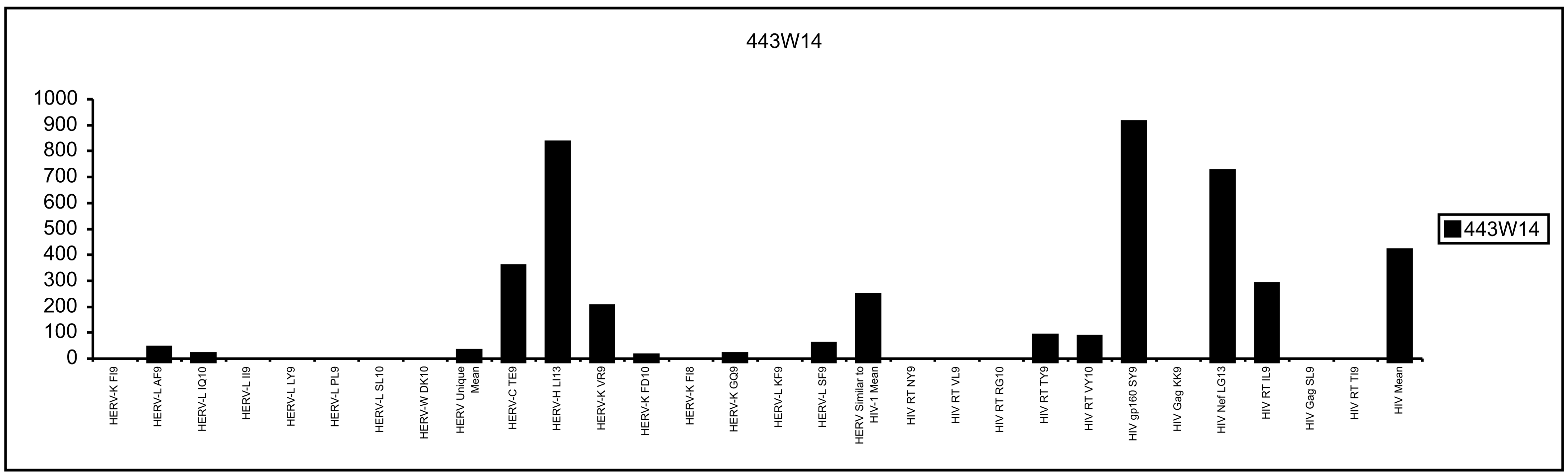
443W14
1000
900
800
700
600
500
400
300
200
100
0
443W14
HERV-K FI9
HERV-L AF9
HERV-L IQ10
HERV-L II9
HERV-L LY9
HERV-L PL9
HERV-L SL10
HERV-W DK10
HERV Unique Mean
HERV-C TE9
HERV-H LI13
HERV-K VR9
HERV-K FD10
HERV-K FI8
HERV-K GQ9
HERV-L KF9
HERV-L SF9
HERV Similar to HIV-1 Mean
HIV RT NY9
HIV RT VL9
HIV RT RG10
HIV RT TY9
HIV RT VY10
HIV gp160 SY9
HIV Gag KK9
HIV Nef LG13
HIV RT IL9
HIV Gag SL9
HIV RT TI9
HIV Mean

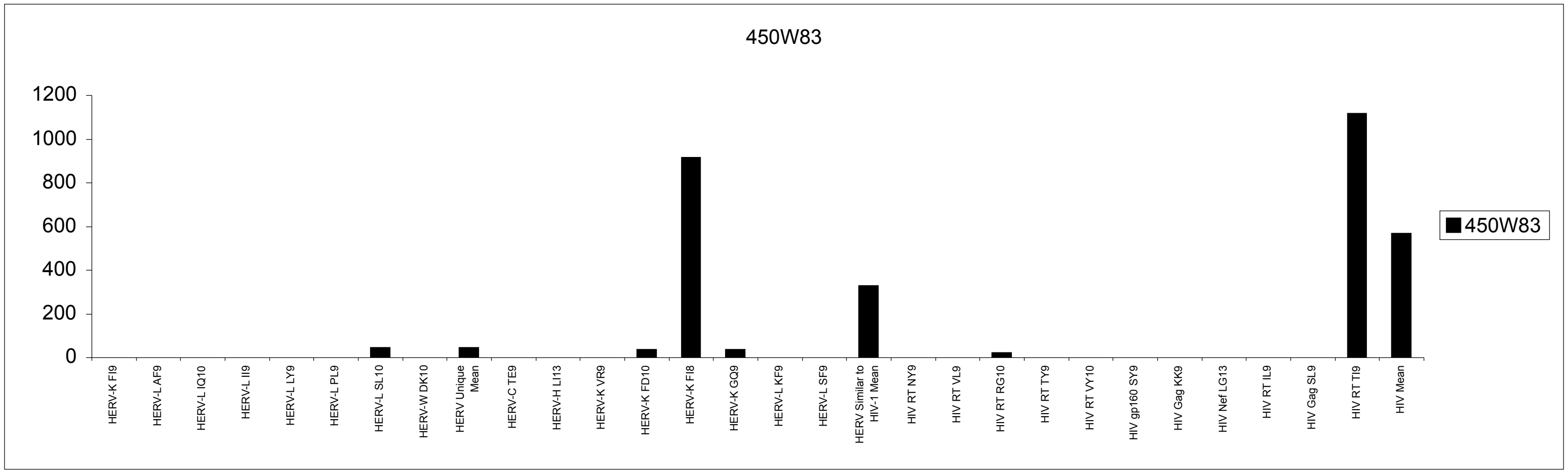

450W83
1200
1000
800
600
400
200
0
450W83
HERV-K FI9
HERV-L AF9
HERV-L IQ10
HERV-L II9
HERV-L LY9
HERV-L PL9
HERV-L SL10
HERV-W DK10
HERV Unique Mean
HERV-C TE9
HERV-H LI13
HERV-K VR9
HERV-K FD10
HERV-K FI8
HERV-K GQ9
HERV-L KF9
HERV-L SF9
HERV Similar to HIV-1 Mean
HIV RT NY9
HIV RT VL9
HIV RT RG10
HIV RT TY9
HIV RT VY10
HIV gp160 SY9
HIV Gag KK9
HIV Nef LG13
HIV RT IL9
HIV Gag SL9
HIV RT TI9
HIV Mean

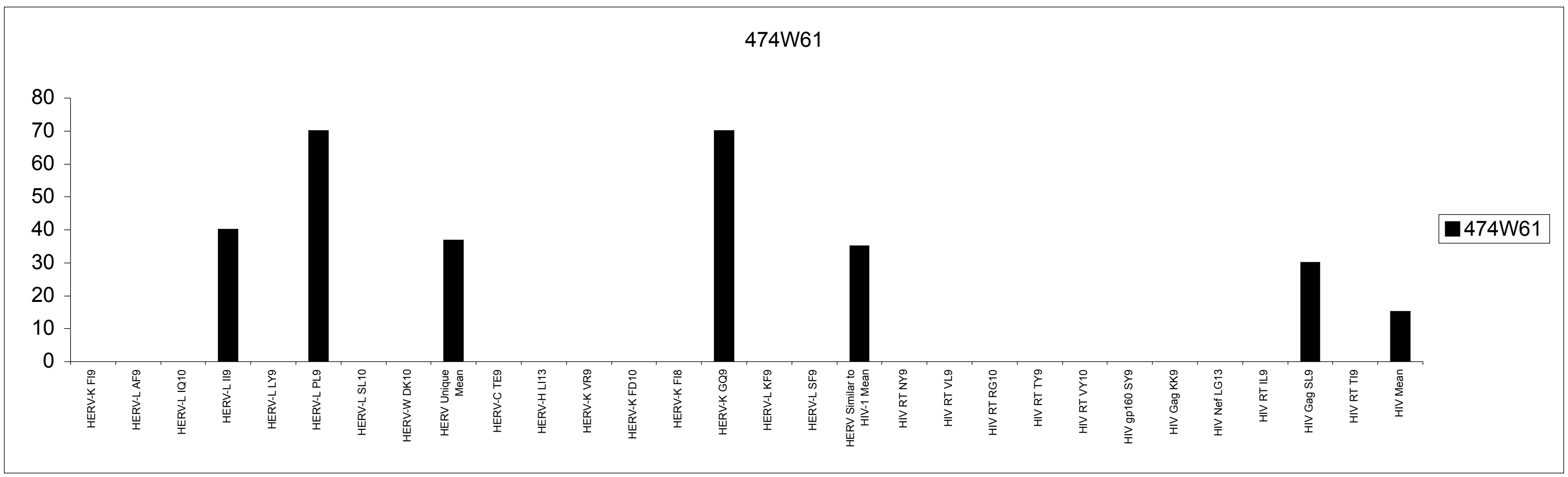

474W61
80
70
60
50
40
30
20
10
0
474W61
HERV-K FI9
HERV-L AF9
HERV-L IQ10
HERV-L II9
HERV-L LY9
HERV-L PL9
HERV-L SL10
HERV-W DK10
HERV Unique Mean
HERV-C TE9
HERV-H LI13
HERV-K VR9
HERV-K FD10
HERV-K FI8
HERV-K GQ9
HERV-L KF9
HERV-L SF9
HERV Similar to HIV-1 Mean
HIV RT NY9
HIV RT VL9
HIV RT RG10
HIV RT TY9
HIV RT VY10
HIV gp160 SY9
HIV Gag KK9
HIV Nef LG13
HIV RT IL9
HIV Gag SL9
HIV RT TI9
HIV Mean

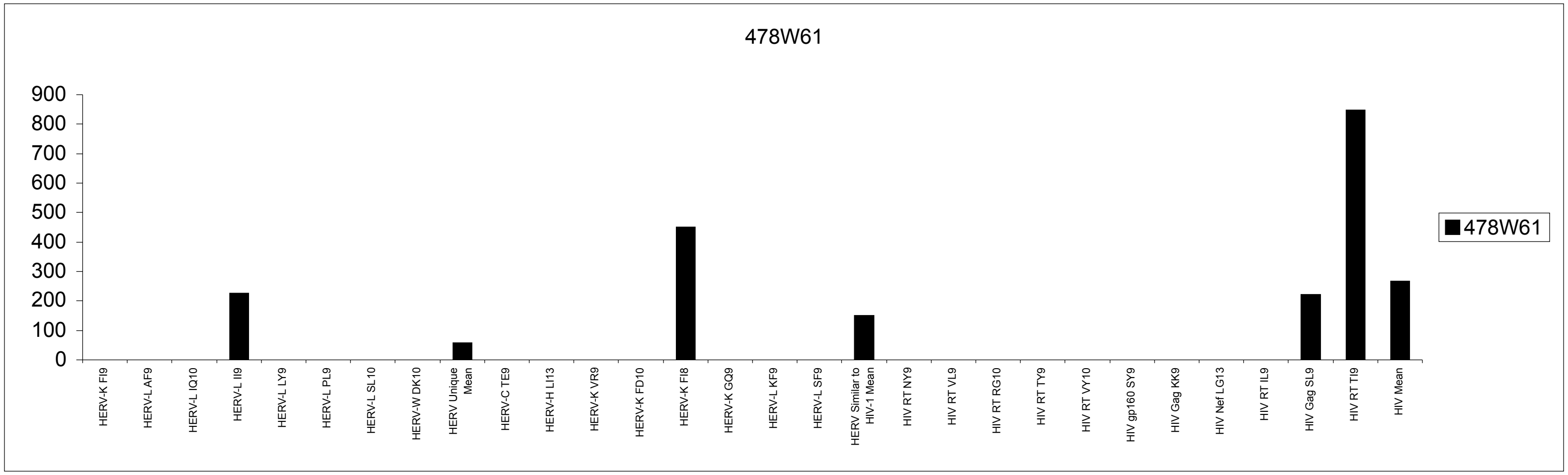
478W61
900
800
700
600
500
400
300
200
100
0
HERV-K FI9
HERV-L AF9
HERV-L IQ10
HERV-L II9
HERV-L LY9
HERV-L PL9
HERV-L SL10
HERV-W DK10
HERV Unique Mean
HERV-C TE9
HERV-H LI13
HERV-K VR9
HERV-K FD10
HERV-K FI8
HERV-K GQ9
HERV-L KF9
HERV-L SF9
HERV Similar to HIV-1 Mean
HIV RT NY9
HIV RT VL9
HIV RT RG10
HIV RT TY9
HIV RT VY10
HIV gp160 SY9
HIV Gag KK9
HIV Nef LG13
HIV RT IL9
HIV Gag SL9
HIV RT TI9
HIV Mean
478W61

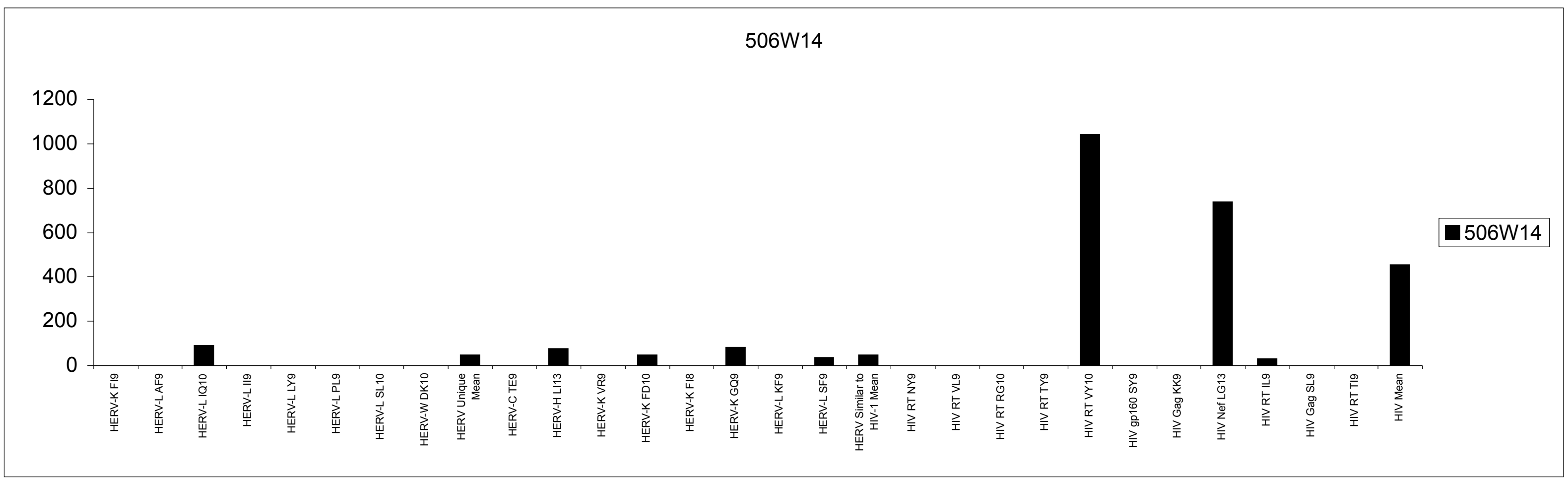
506W14
1200
1000
800
600
400
200
0
HERV-K FI9
HERV-L AF9
HERV-L IQ10
HERV-L II9
HERV-L LY9
HERV-L PL9
HERV-L SL10
HERV-W DK10
HERV Unique Mean
HERV-C TE9
HERV-H LI13
HERV-K VR9
HERV-K FD10
HERV-K FI8
HERV-K GQ9
HERV-L KF9
HERV-L SF9
HERV Similar to HIV-1 Mean
HIV RT NY9
HIV RT VL9
HIV RT RG10
HIV RT TY9
HIV RT VY10
HIV gp160 SY9
HIV Gag KK9
HIV Nef LG13
HIV RT IL9
HIV Gag SL9
HIV RT TI9
HIV Mean
506W14

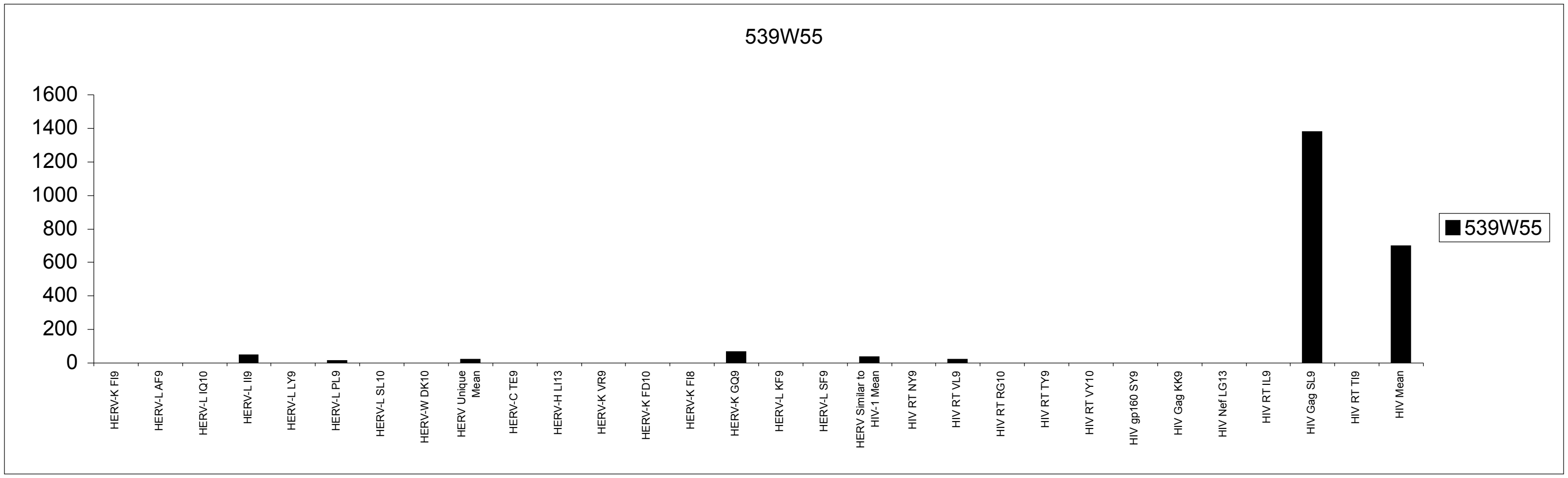

539W55
1600
1400
1200
1000
800
600
400
200
0
HERV-K FI9
HERV-L AF9
HERV-L IQ10
HERV-L II9
HERV-L LY9
HERV-L PL9
HERV-L SL10
HERV-W DK10
HERV Unique Mean
HERV-C TE9
HERV-H LI13
HERV-K VR9
HERV-K FD10
HERV-K FI8
HERV-K GQ9
HERV-L KF9
HERV-L SF9
HERV Similar to HIV-1 Mean
HIV RT NY9
HIV RT VL9
HIV RT RG10
HIV RT TY9
HIV RT VY10
HIV gp160 SY9
HIV Gag KK9
HIV Nef LG13
HIV RT IL9
HIV Gag SL9
HIV RT TI9
HIV Mean
539W55

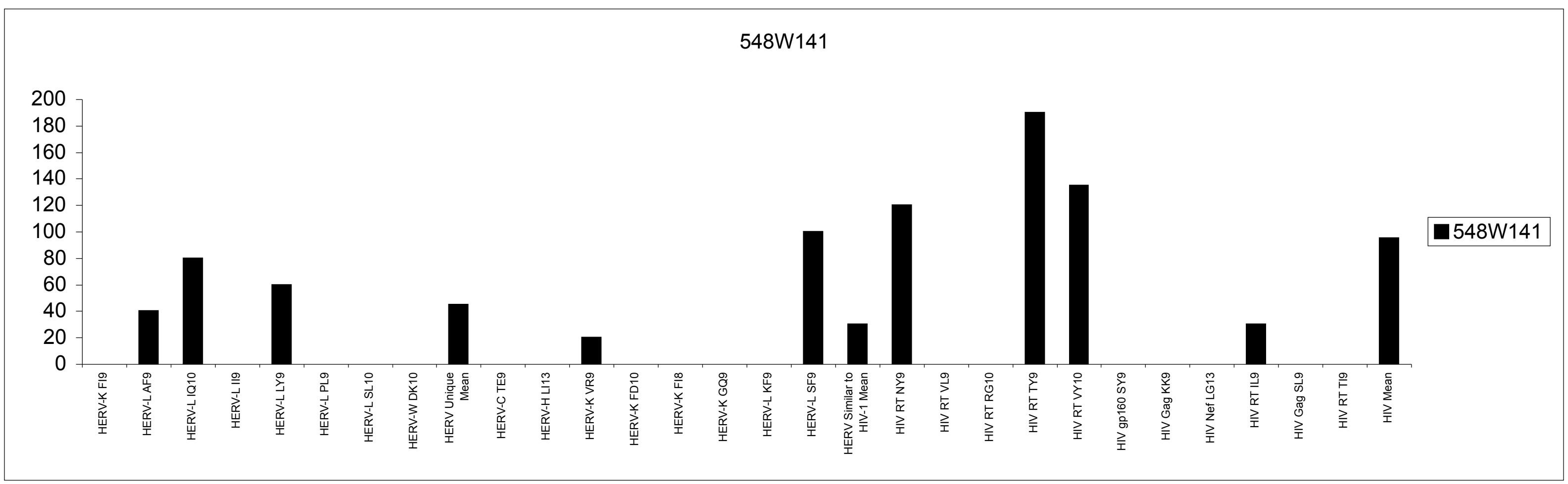

548W141
200
180
160
140
120
100
80
60
40
20
0
HERV-K FI9
HERV-L AF9
HERV-L IQ10
HERV-L II9
HERV-L LY9
HERV-L PL9
HERV-L SL10
HERV-W DK10
HERV Unique Mean
HERV-C TE9
HERV-H LI13
HERV-K VR9
HERV-K FD10
HERV-K FI8
HERV-K GQ9
HERV-L KF9
HERV-L SF9
HERV Similar to HIV-1 Mean
HIV RT NY9
HIV RT VL9
HIV RT RG10
HIV RT TY9
HIV RT VY10
HIV gp160 SY9
HIV Gag KK9
HIV Nef LG13
HIV RT IL9
HIV Gag SL9
HIV RT TI9
HIV Mean
548W141

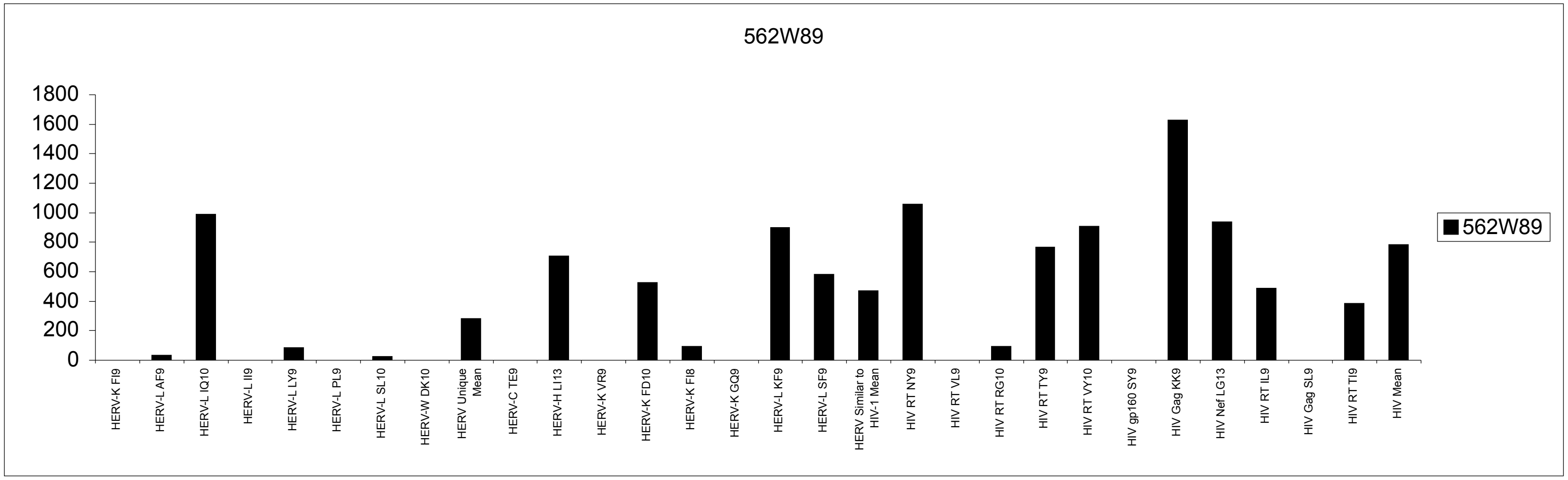
562W89
1800
1600
1400
1200
1000
800
600
400
200
0
562W89
HERV-K FI9
HERV-L AF9
HERV-L IQ10
HERV-L II9
HERV-L LY9
HERV-L PL9
HERV-L SL10
HERV-W DK10
HERV Unique Mean
HERV-C TE9
HERV-H LI13
HERV-K VR9
HERV-K FD10
HERV-K FI8
HERV-K GQ9
HERV-L KF9
HERV-L SF9
HERV Similar to HIV-1 Mean
HIV RT NY9
HIV RT VL9
HIV RT RG10
HIV RT TY9
HIV RT VY10
HIV gp160 SY9
HIV Gag KK9
HIV Nef LG13
HIV RT IL9
HIV Gag SL9
HIV RT TI9
HIV Mean

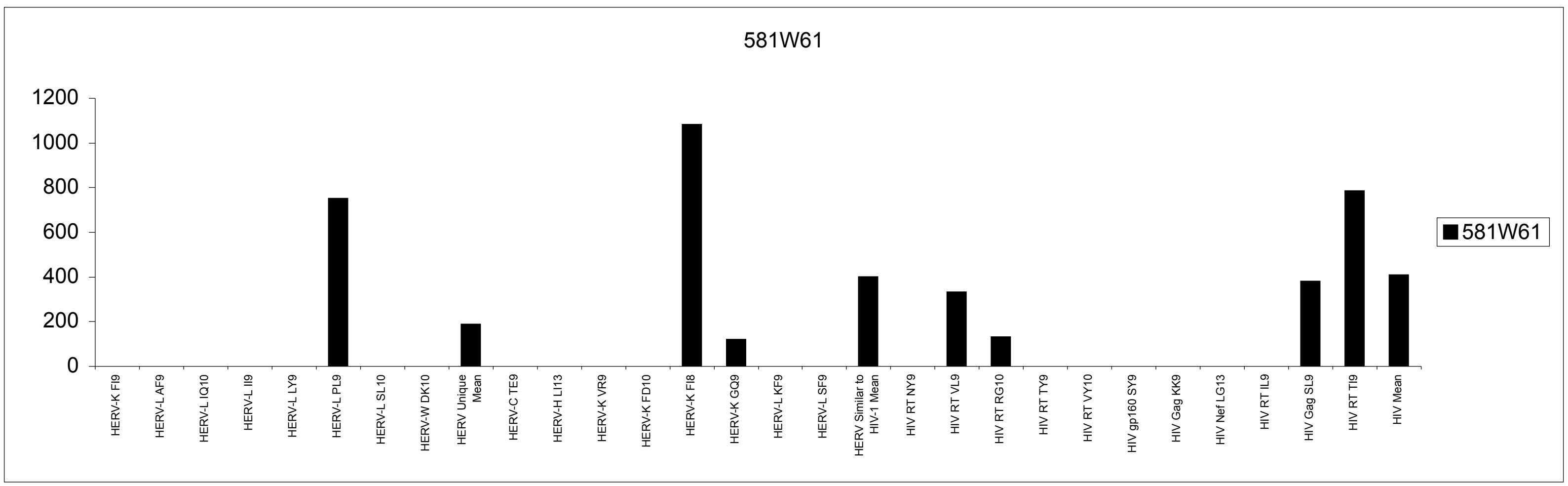
581W61
1200
1000
800
600
400
200
0
581W61
HERV-K FI9
HERV-L AF9
HERV-L IQ10
HERV-L II9
HERV-L LY9
HERV-L PL9
HERV-L SL10
HERV-W DK10
HERV Unique Mean
HERV-C TE9
HERV-H LI13
HERV-K VR9
HERV-K FD10
HERV-K FI8
HERV-K GQ9
HERV-L KF9
HERV-L SF9
HERV Similar to HIV-1 Mean
HIV RT NY9
HIV RT VL9
HIV RT RG10
HIV RT TY9
HIV RT VY10
HIV gp160 SY9
HIV Gag KK9
HIV Nef LG13
HIV RT IL9
HIV Gag SL9
HIV RT TI9
HIV Mean

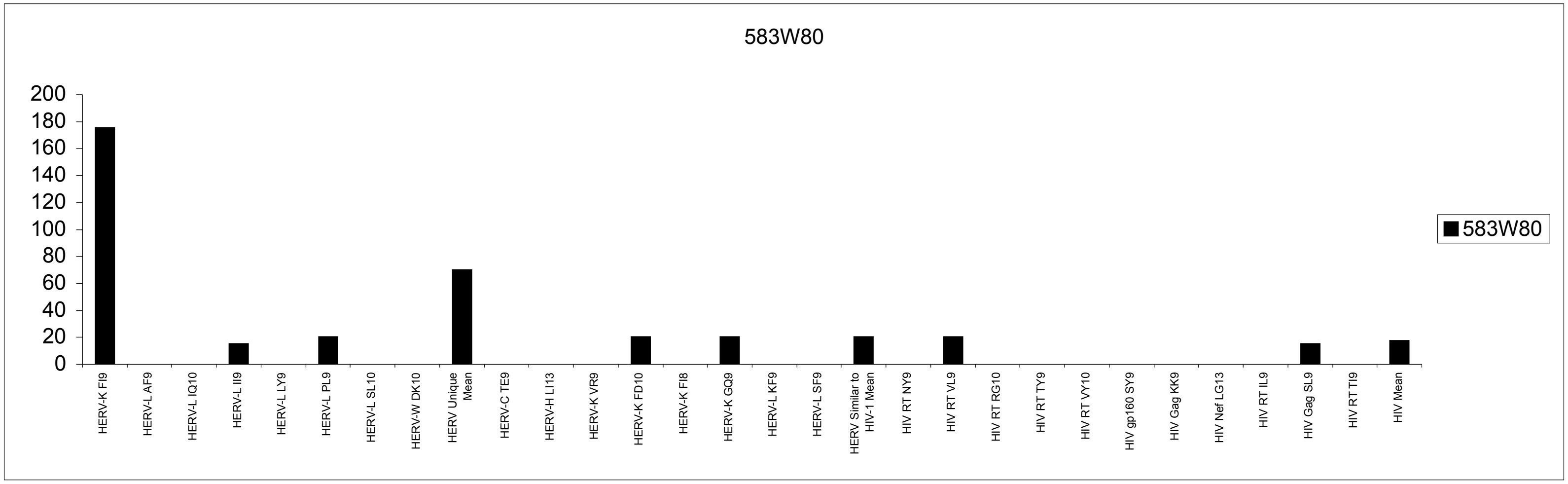
583W80
200
180
160
140
120
100
80
60
40
20
0
HERV-K FI9
HERV-L AF9
HERV-L IQ10
HERV-L II9
HERV-L LY9
HERV-L PL9
HERV-L SL10
HERV-W DK10
HERV Unique Mean
HERV-C TE9
HERV-H LI13
HERV-K VR9
HERV-K FD10
HERV-K FI8
HERV-K GQ9
HERV-L KF9
HERV-L SF9
HERV Similar to HIV-1 Mean
HIV RT NY9
HIV RT VL9
HIV RT RG10
HIV RT TY9
HIV RT VY10
HIV gp160 SY9
HIV Gag KK9
HIV Nef LG13
HIV RT IL9
HIV Gag SL9
HIV RT TI9
HIV Mean
583W80

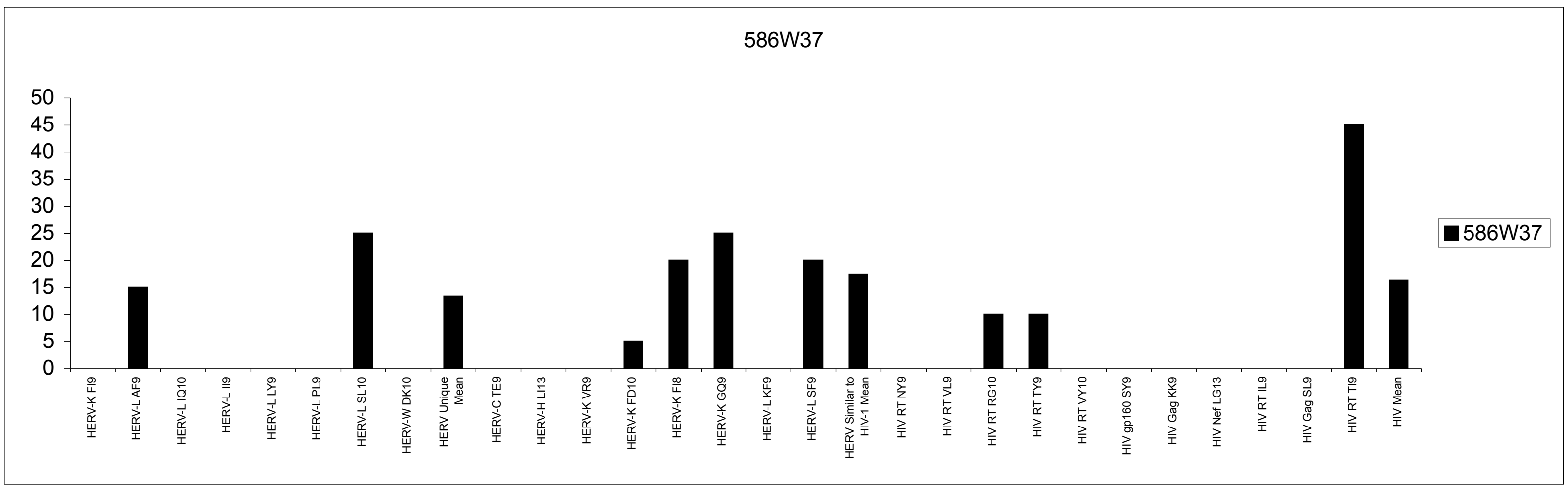
586W37
50
45
40
35
30
25
20
15
10
5
0
HERV-K FI9
HERV-L AF9
HERV-L IQ10
HERV-L II9
HERV-L LY9
HERV-L PL9
HERV-L SL10
HERV-W DK10
HERV Unique Mean
HERV-C TE9
HERV-H LI13
HERV-K VR9
HERV-K FD10
HERV-K FI8
HERV-K GQ9
HERV-L KF9
HERV-L SF9
HERV Similar to HIV-1 Mean
HIV RT NY9
HIV RT VL9
HIV RT RG10
HIV RT TY9
HIV RT VY10
HIV gp160 SY9
HIV Gag KK9
HIV Nef LG13
HIV RT IL9
HIV Gag SL9
HIV RT TI9
HIV Mean
586W37

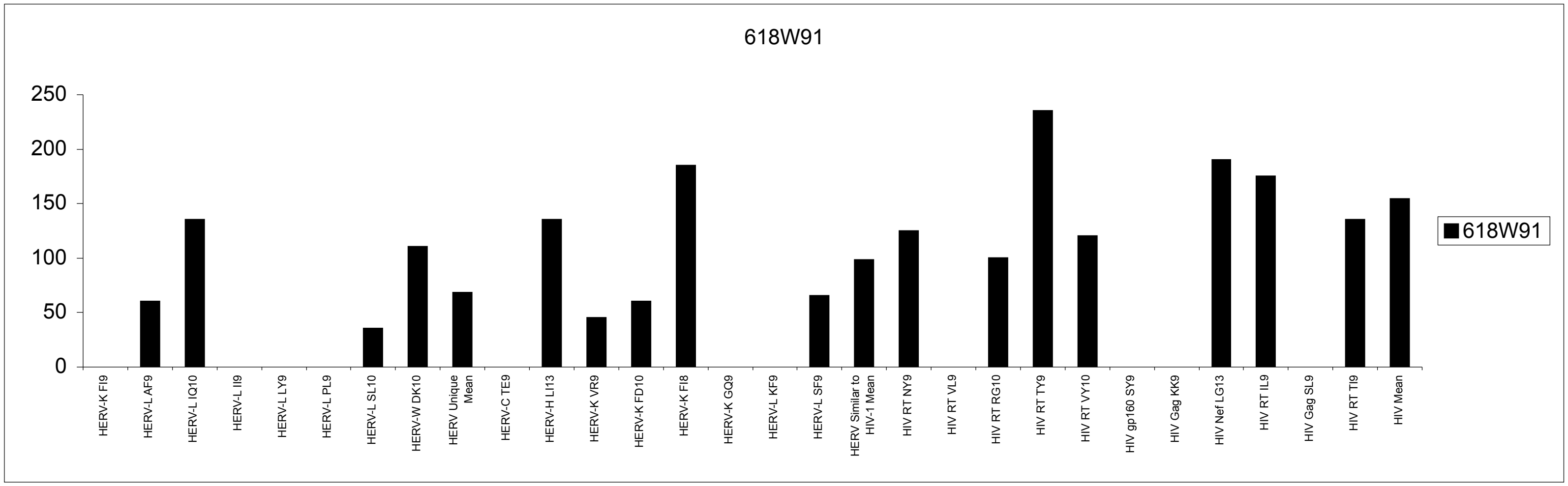
618W91
250
200
150
100
50
0
HERV-K FI9
HERV-L AF9
HERV-L IQ10
HERV-L II9
HERV-L LY9
HERV-L PL9
HERV-L SL10
HERV-W DK10
HERV Unique Mean
HERV-C TE9
HERV-H LI13
HERV-K VR9
HERV-K FD10
HERV-K FI8
HERV-K GQ9
HERV-L KF9
HERV-L SF9
HERV Similar to HIV-1 Mean
HIV RT NY9
HIV RT VL9
HIV RT RG10
HIV RT TY9
HIV RT VY10
HIV gp160 SY9
HIV Gag KK9
HIV Nef LG13
HIV RT IL9
HIV Gag SL9
HIV RT TI9
HIV Mean
618W91

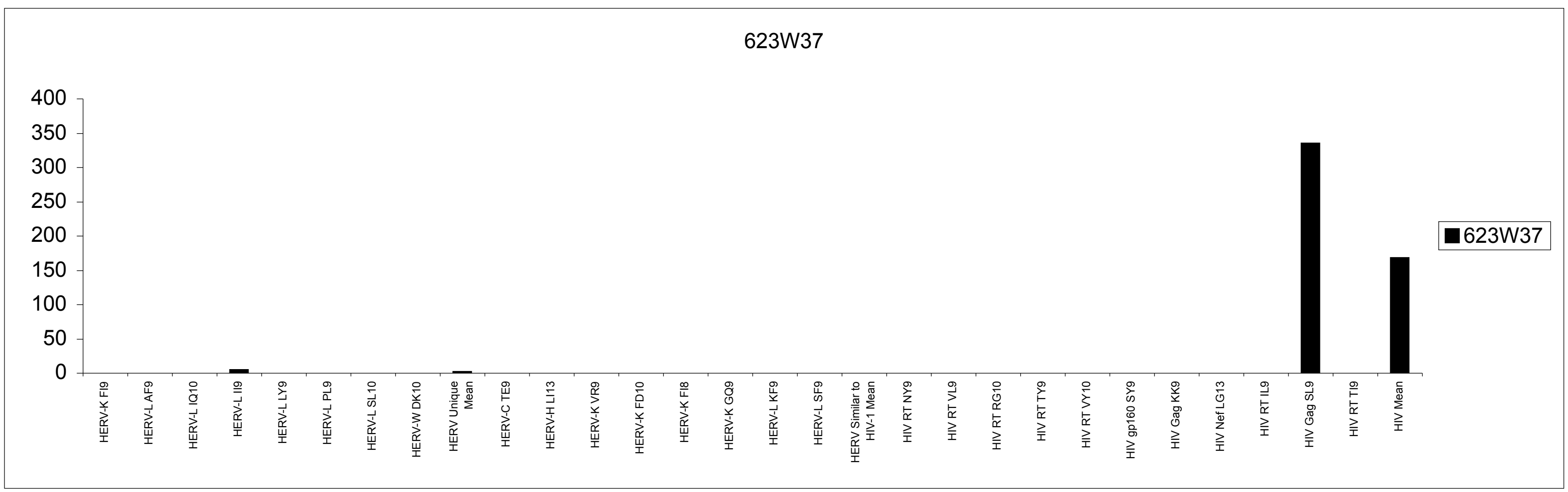
623W37
400
350
300
250
200
150
100
50
0
HERV-K FI9
HERV-L AF9
HERV-L IQ10
HERV-L II9
HERV-L LY9
HERV-L PL9
HERV-L SL10
HERV-W DK10
HERV Unique Mean
HERV-C TE9
HERV-H LI13
HERV-K VR9
HERV-K FD10
HERV-K FI8
HERV-K GQ9
HERV-L KF9
HERV-L SF9
HERV Similar to HIV-1 Mean
HIV RT NY9
HIV RT VL9
HIV RT RG10
HIV RT TY9
HIV RT VY10
HIV gp160 SY9
HIV Gag KK9
HIV Nef LG13
HIV RT IL9
HIV Gag SL9
HIV RT TI9
HIV Mean
623W37

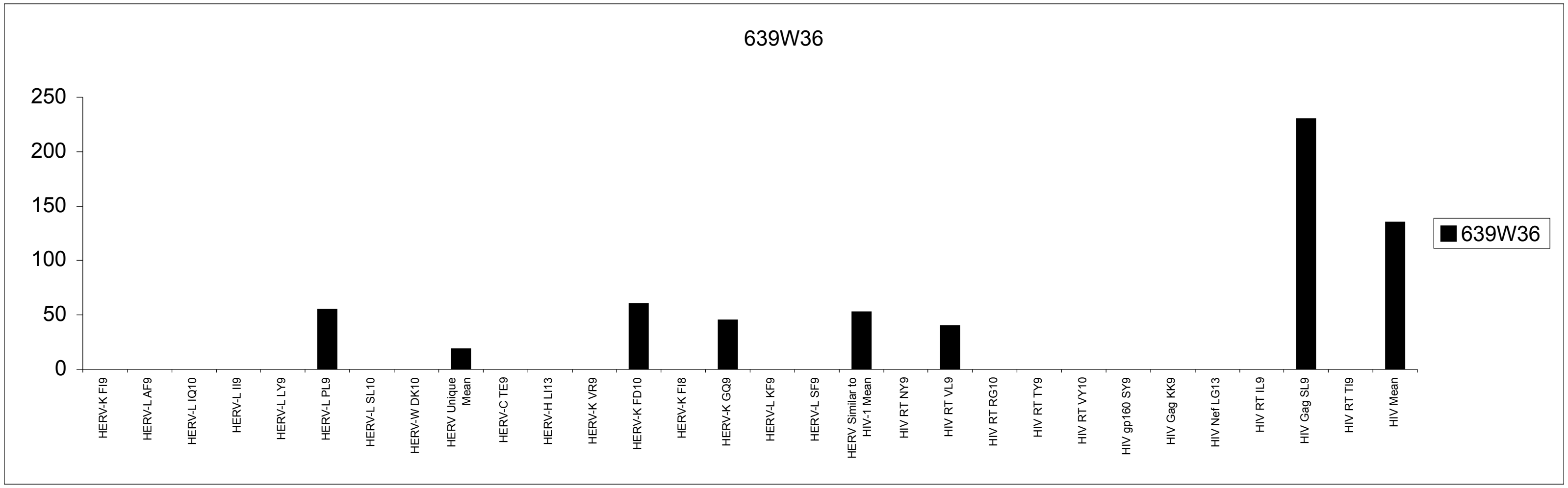
639W36
250
200
150
100
50
0
HERV-K FI9
HERV-L AF9
HERV-L IQ10
HERV-L II9
HERV-L LY9
HERV-L PL9
HERV-L SL10
HERV-W DK10
HERV Unique Mean
HERV-C TE9
HERV-H LI13
HERV-K VR9
HERV-K FD10
HERV-K FI8
HERV-K GQ9
HERV-L KF9
HERV-L SF9
HERV Similar to HIV-1 Mean
HIV RT NY9
HIV RT VL9
HIV RT RG10
HIV RT TY9
HIV RT VY10
HIV gp160 SY9
HIV Gag KK9
HIV Nef LG13
HIV RT IL9
HIV Gag SL9
HIV RT TI9
HIV Mean
639W36

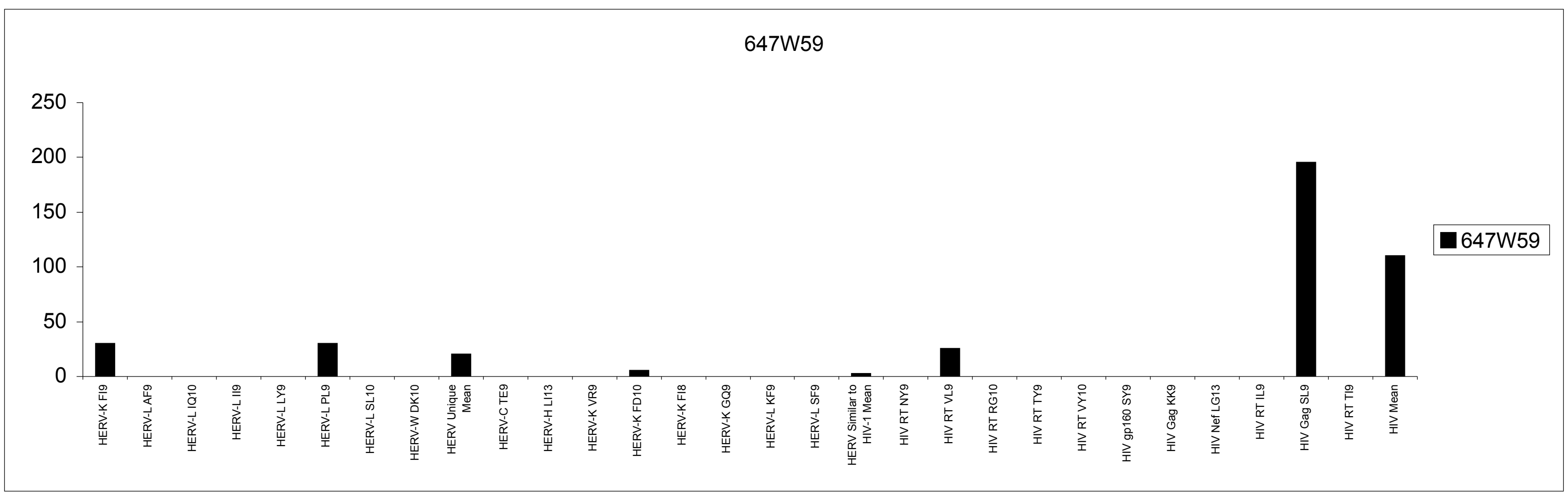
647W59
250
200
150
100
50
0
HERV-K FI9
HERV-L AF9
HERV-L IQ10
HERV-L II9
HERV-L LY9
HERV-L PL9
HERV-L SL10
HERV-W DK10
HERV Unique Mean
HERV-C TE9
HERV-H LI13
HERV-K VR9
HERV-K FD10
HERV-K FI8
HERV-K GQ9
HERV-L KF9
HERV-L SF9
HERV Similar to HIV-1 Mean
HIV RT NY9
HIV RT VL9
HIV RT RG10
HIV RT TY9
HIV RT VY10
HIV gp160 SY9
HIV Gag KK9
HIV Nef LG13
HIV RT IL9
HIV Gag SL9
HIV RT TI9
HIV Mean
647W59

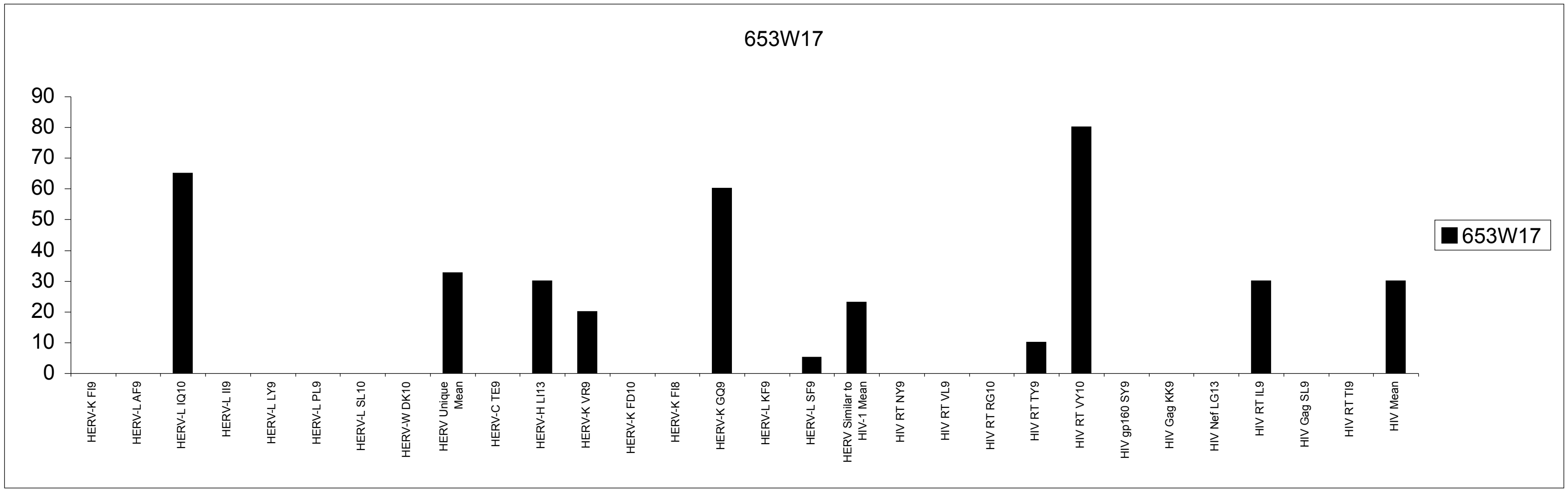

653W17
90
80
70
60
50
40
30
20
10
0
HERV-K FI9
HERV-L AF9
HERV-L IQ10
HERV-L II9
HERV-L LY9
HERV-L PL9
HERV-L SL10
HERV-W DK10
HERV Unique Mean
HERV-C TE9
HERV-H LI13
HERV-K VR9
HERV-K FD10
HERV-K FI8
HERV-K GQ9
HERV-L KF9
HERV-L SF9
HERV Similar to HIV-1 Mean
HIV RT NY9
HIV RT VL9
HIV RT RG10
HIV RT TY9
HIV RT VY10
HIV gp160 SY9
HIV Gag KK9
HIV Nef LG13
HIV RT IL9
HIV Gag SL9
HIV RT TI9
HIV Mean
653W17

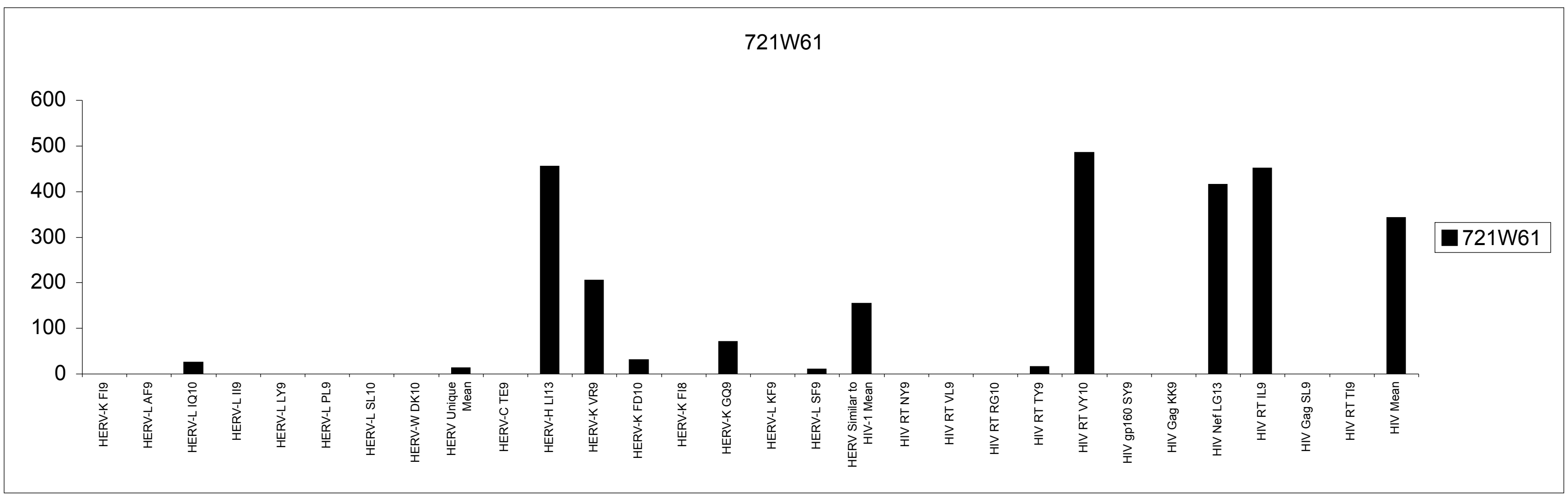

721W61
600
500
400
300
200
100
0
HERV-K FI9
HERV-L AF9
HERV-L IQ10
HERV-L II9
HERV-L LY9
HERV-L PL9
HERV-L SL10
HERV-W DK10
HERV Unique Mean
HERV-C TE9
HERV-H LI13
HERV-K VR9
HERV-K FD10
HERV-K FI8
HERV-K GQ9
HERV-L KF9
HERV-L SF9
HERV Similar to HIV-1 Mean
HIV RT NY9
HIV RT VL9
HIV RT RG10
HIV RT TY9
HIV RT VY10
HIV gp160 SY9
HIV Gag KK9
HIV Nef LG13
HIV RT IL9
HIV Gag SL9
HIV RT TI9
HIV Mean
721W61

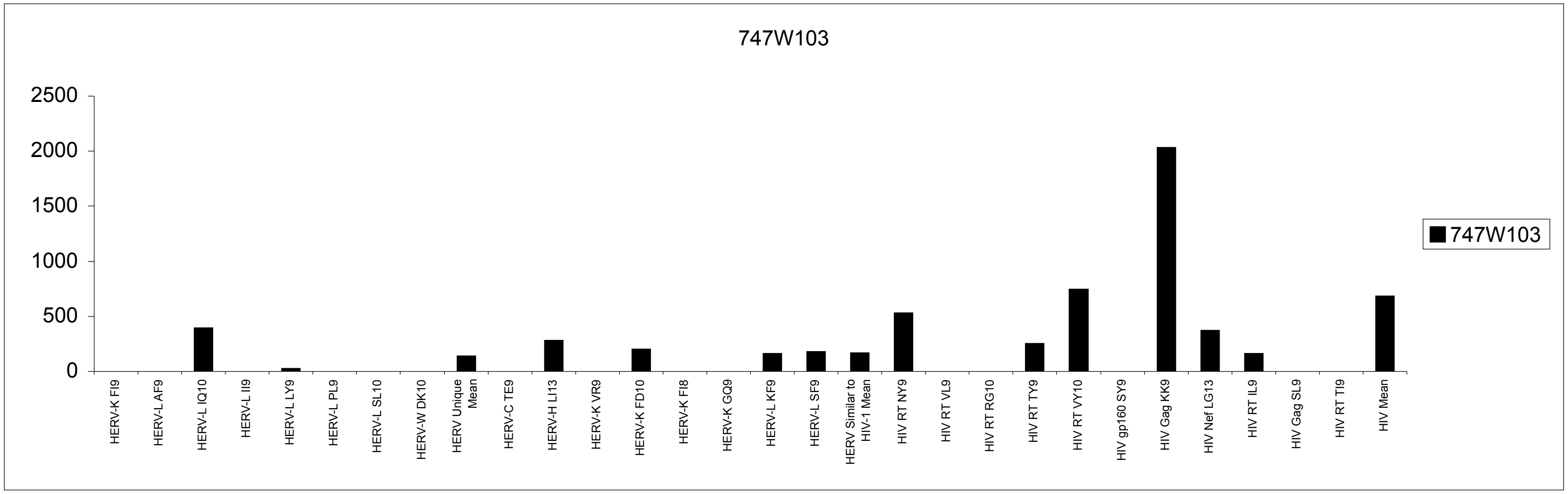

747W103
2500
2000
1500
1000
500
0
HERV-K FI9
HERV-L AF9
HERV-L IQ10
HERV-L II9
HERV-L LY9
HERV-L PL9
HERV-L SL10
HERV-W DK10
HERV Unique Mean
HERV-C TE9
HERV-H LI13
HERV-K VR9
HERV-K FD10
HERV-K FI8
HERV-K GQ9
HERV-L KF9
HERV-L SF9
HERV Similar to HIV-1 Mean
HIV RT NY9
HIV RT VL9
HIV RT RG10
HIV RT TY9
HIV RT VY10
HIV gp160 SY9
HIV Gag KK9
HIV Nef LG13
HIV RT IL9
HIV Gag SL9
HIV RT TI9
HIV Mean
747W103

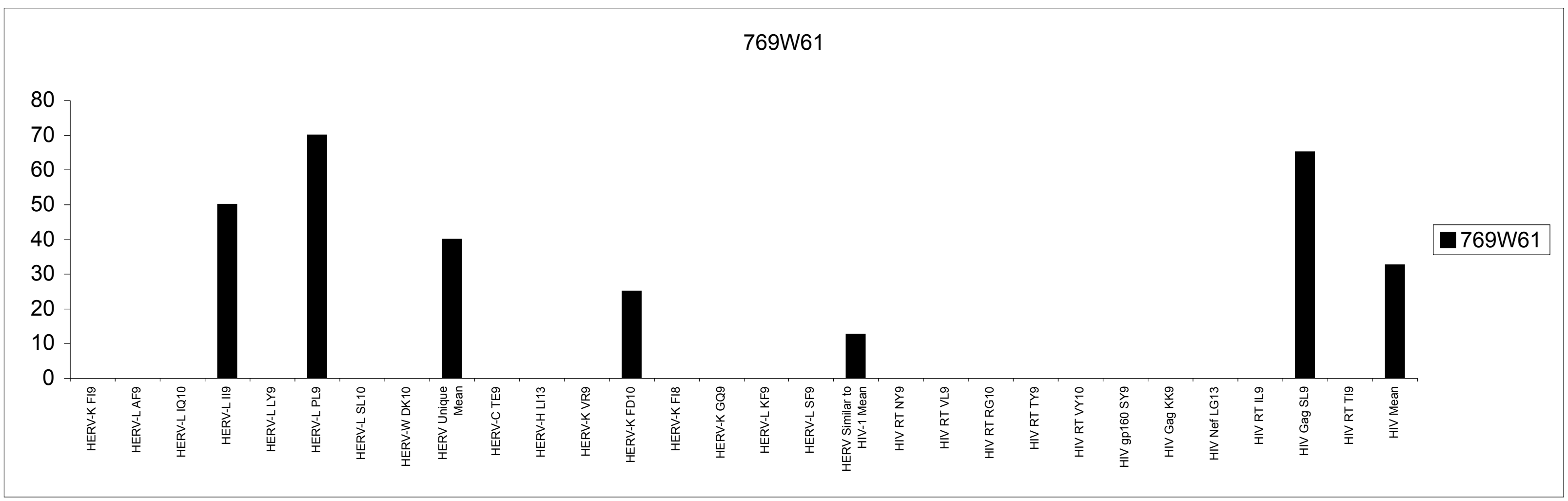

769W61
80
70
60
50
40
30
20
10
0
HERV-K FI9
HERV-L AF9
HERV-L IQ10
HERV-L II9
HERV-L LY9
HERV-L PL9
HERV-L SL10
HERV-W DK10
HERV Unique Mean
HERV-C TE9
HERV-H LI13
HERV-K VR9
HERV-K FD10
HERV-K FI8
HERV-K GQ9
HERV-L KF9
HERV-L SF9
HERV Similar to HIV-1 Mean
HIV RT NY9
HIV RT VL9
HIV RT RG10
HIV RT TY9
HIV RT VY10
HIV gp160 SY9
HIV Gag KK9
HIV Nef LG13
HIV RT IL9
HIV Gag SL9
HIV RT TI9
HIV Mean
769W61

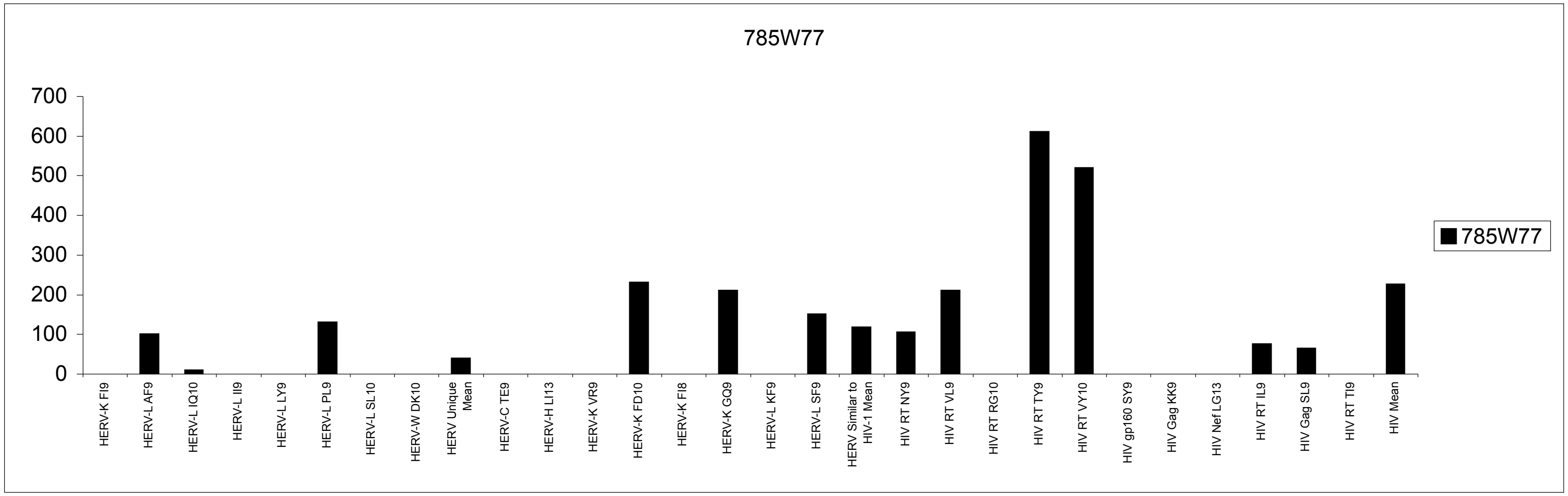

785W77
700
600
500
400
300
200
100
0
HERV-K FI9
HERV-L AF9
HERV-L IQ10
HERV-L II9
HERV-L LY9
HERV-L PL9
HERV-L SL10
HERV-W DK10
HERV Unique Mean
HERV-C TE9
HERV-H LI13
HERV-K VR9
HERV-K FD10
HERV-K FI8
HERV-K GQ9
HERV-L KF9
HERV-L SF9
HERV Similar to HIV-1 Mean
HIV RT NY9
HIV RT VL9
HIV RT RG10
HIV RT TY9
HIV RT VY10
HIV gp160 SY9
HIV Gag KK9
HIV Nef LG13
HIV RT IL9
HIV Gag SL9
HIV RT TI9
HIV Mean
785W77

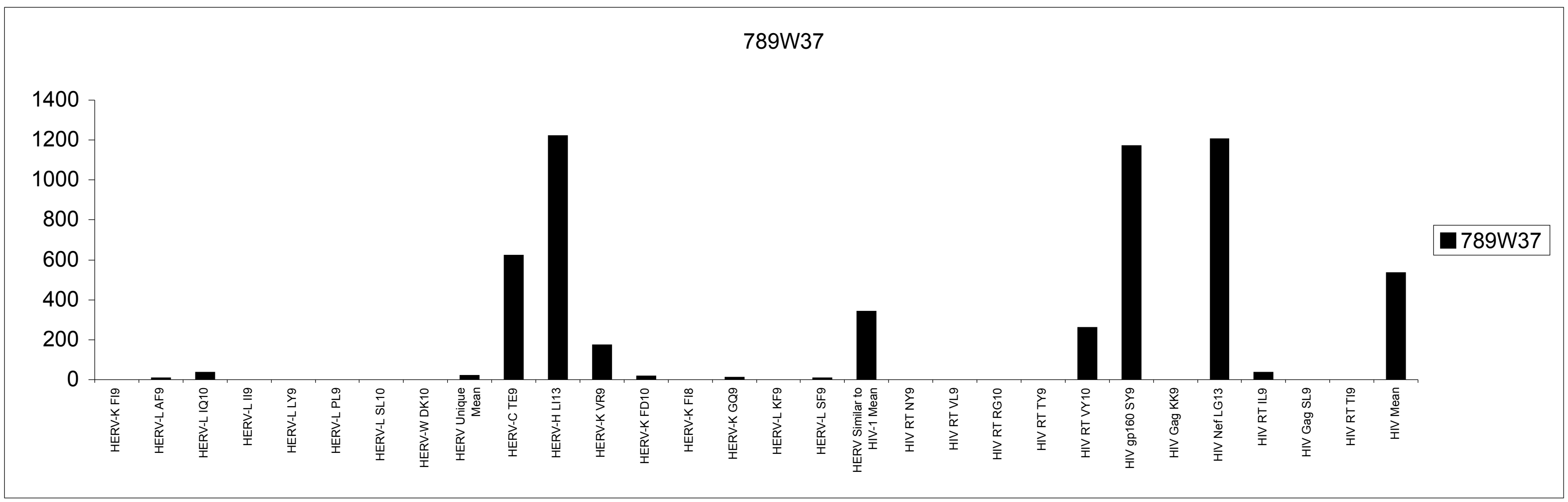

789W37
1400
1200
1000
800
600
400
200
0
HERV-K FI9
HERV-L AF9
HERV-L IQ10
HERV-L II9
HERV-L LY9
HERV-L PL9
HERV-L SL10
HERV-W DK10
HERV Unique Mean
HERV-C TE9
HERV-H LI13
HERV-K VR9
HERV-K FD10
HERV-K FI8
HERV-K GQ9
HERV-L KF9
HERV-L SF9
HERV Similar to HIV-1 Mean
HIV RT NY9
HIV RT VL9
HIV RT RG10
HIV RT TY9
HIV RT VY10
HIV gp160 SY9
HIV Gag KK9
HIV Nef LG13
HIV RT IL9
HIV Gag SL9
HIV RT TI9
HIV Mean
789W37

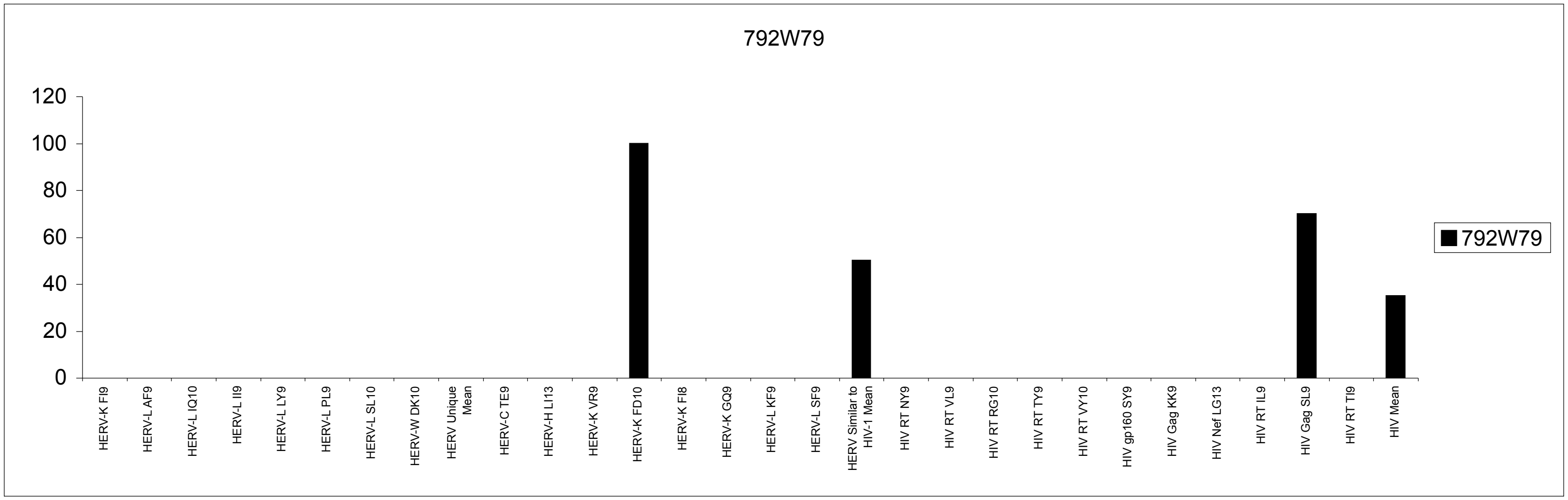
792W79
120
100
80
60
40
20
0
HERV-K FI9
HERV-L AF9
HERV-L IQ10
HERV-L II9
HERV-L LY9
HERV-L PL9
HERV-L SL10
HERV-W DK10
HERV Unique Mean
HERV-C TE9
HERV-H LI13
HERV-K VR9
HERV-K FD10
HERV-K FI8
HERV-K GQ9
HERV-L KF9
HERV-L SF9
HERV Similar to HIV-1 Mean
HIV RT NY9
HIV RT VL9
HIV RT RG10
HIV RT TY9
HIV RT VY10
HIV gp160 SY9
HIV Gag KK9
HIV Nef LG13
HIV RT IL9
HIV Gag SL9
HIV RT TI9
HIV Mean
792W79

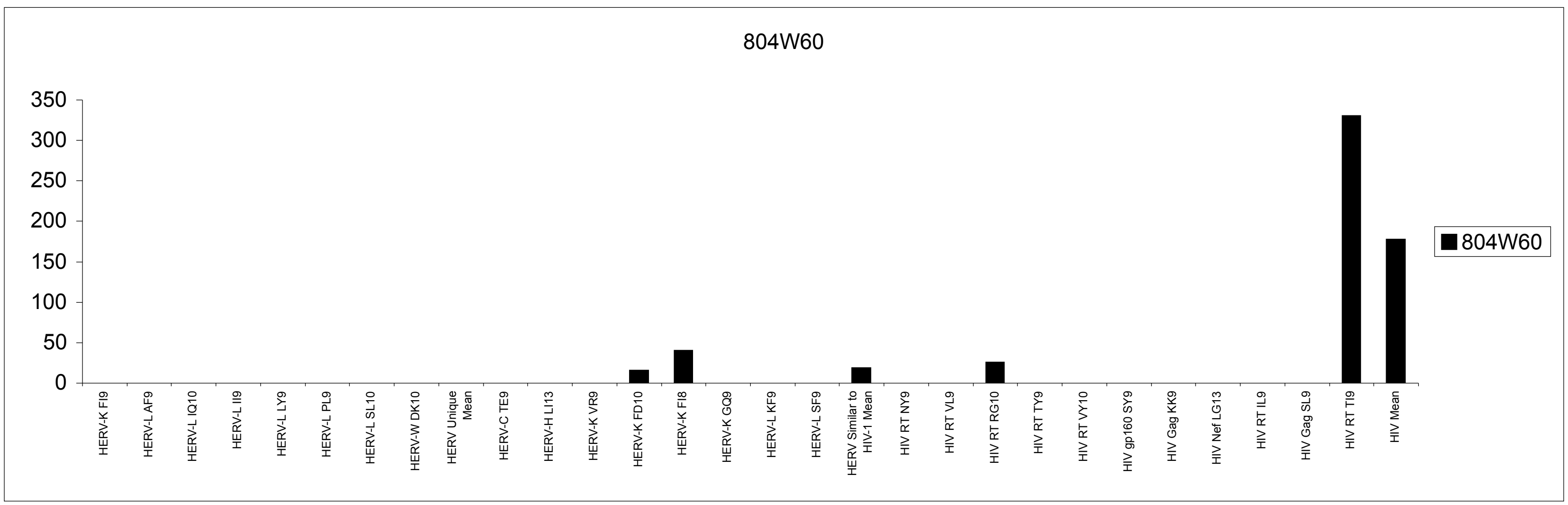
804W60
350
300
250
200
150
100
50
0
HERV-K FI9
HERV-L AF9
HERV-L IQ10
HERV-L II9
HERV-L LY9
HERV-L PL9
HERV-L SL10
HERV-W DK10
HERV Unique Mean
HERV-C TE9
HERV-H LI13
HERV-K VR9
HERV-K FD10
HERV-K FI8
HERV-K GQ9
HERV-L KF9
HERV-L SF9
HERV Similar to HIV-1 Mean
HIV RT NY9
HIV RT VL9
HIV RT RG10
HIV RT TY9
HIV RT VY10
HIV gp160 SY9
HIV Gag KK9
HIV Nef LG13
HIV RT IL9
HIV Gag SL9
HIV RT TI9
HIV Mean
804W60

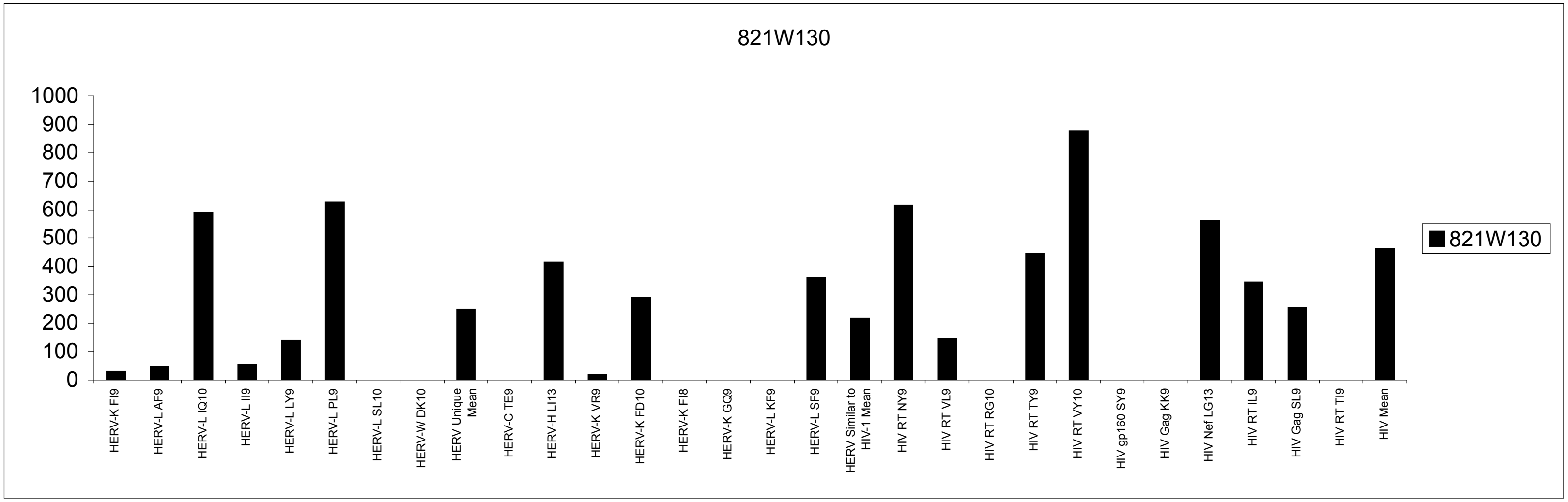
821W130
1000
900
800
700
600
500
400
300
200
100
0
HERV-K FI9
HERV-L AF9
HERV-L IQ10
HERV-L II9
HERV-L LY9
HERV-L PL9
HERV-L SL10
HERV-W DK10
HERV Unique Mean
HERV-C TE9
HERV-H LI13
HERV-K VR9
HERV-K FD10
HERV-K FI8
HERV-K GQ9
HERV-L KF9
HERV-L SF9
HERV Similar to HIV-1 Mean
HIV RT NY9
HIV RT VL9
HIV RT RG10
HIV RT TY9
HIV RT VY10
HIV gp160 SY9
HIV Gag KK9
HIV Nef LG13
HIV RT IL9
HIV Gag SL9
HIV RT TI9
HIV Mean
821W130

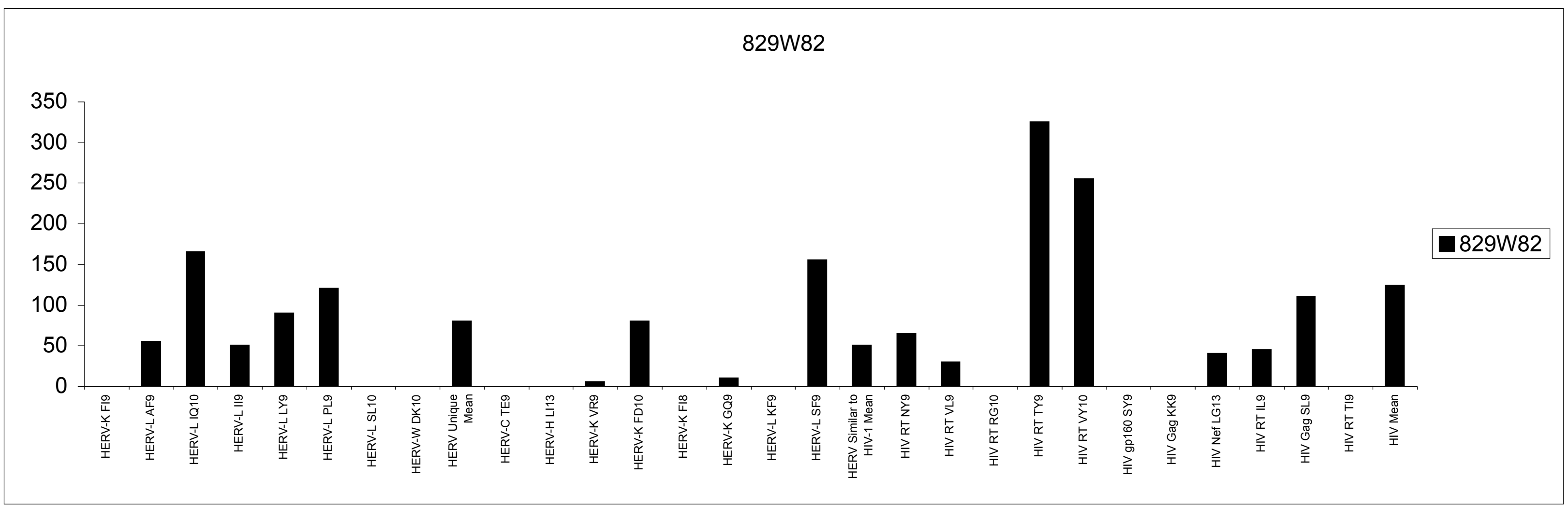
829W82
350
300
250
200
150
100
50
0
HERV-K FI9
HERV-L AF9
HERV-L IQ10
HERV-L II9
HERV-L LY9
HERV-L PL9
HERV-L SL10
HERV-W DK10
HERV Unique Mean
HERV-C TE9
HERV-H LI13
HERV-K VR9
HERV-K FD10
HERV-K FI8
HERV-K GQ9
HERV-L KF9
HERV-L SF9
HERV Similar to HIV-1 Mean
HIV RT NY9
HIV RT VL9
HIV RT RG10
HIV RT TY9
HIV RT VY10
HIV gp160 SY9
HIV Gag KK9
HIV Nef LG13
HIV RT IL9
HIV Gag SL9
HIV RT TI9
HIV Mean
829W82

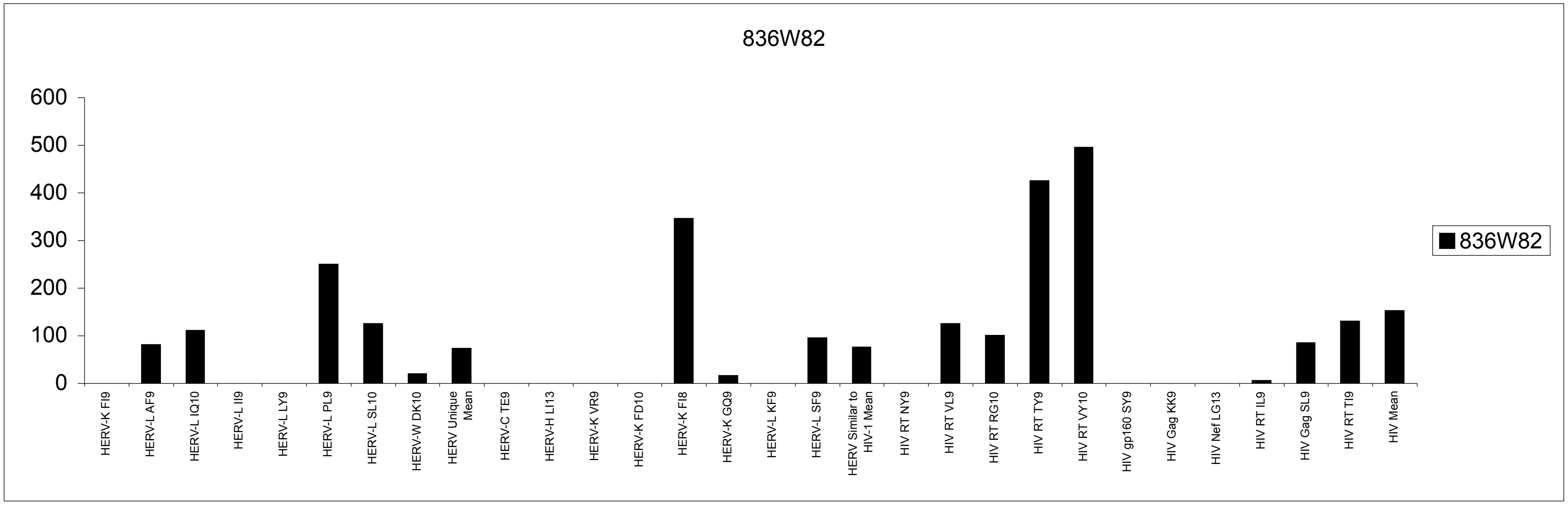
836W82
600
500
400
300
200
100
0
HERV-K FI9
HERV-L AF9
HERV-L IQ10
HERV-L II9
HERV-L LY9
HERV-L PL9
HERV-L SL10
HERV-W DK10
HERV Unique Mean
HERV-C TE9
HERV-H LI13
HERV-K VR9
HERV-K FD10
HERV-K FI8
HERV-K GQ9
HERV-L KF9
HERV-L SF9
HERV Similar to HIV-1 Mean
HIV RT NY9
HIV RT VL9
HIV RT RG10
HIV RT TY9
HIV RT VY10
HIV gp160 SY9
HIV Gag KK9
HIV Nef LG13
HIV RT IL9
HIV Gag SL9
HIV RT TI9
HIV Mean
836W82

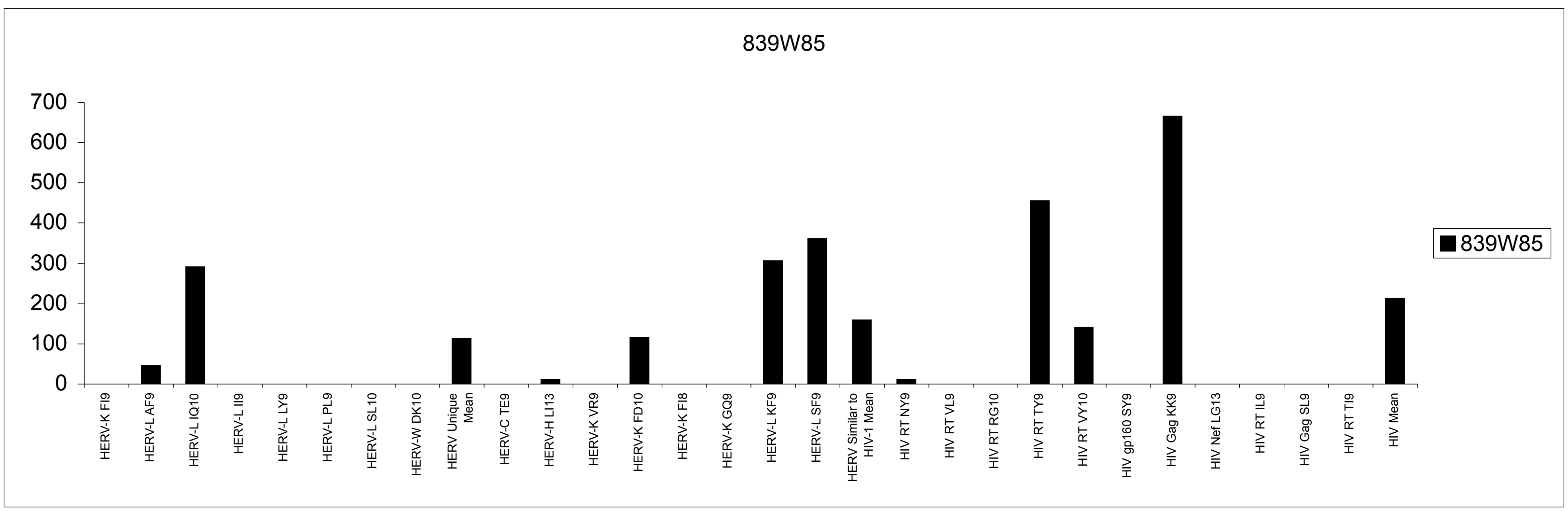
839W85
700
600
500
400
300
200
100
0
HERV-K FI9
HERV-L AF9
HERV-L IQ10
HERV-L II9
HERV-L LY9
HERV-L PL9
HERV-L SL10
HERV-W DK10
HERV Unique Mean
HERV-C TE9
HERV-H LI13
HERV-K VR9
HERV-K FD10
HERV-K FI8
HERV-K GQ9
HERV-L KF9
HERV-L SF9
HERV Similar to HIV-1 Mean
HIV RT NY9
HIV RT VL9
HIV RT RG10
HIV RT TY9
HIV RT VY10
HIV gp160 SY9
HIV Gag KK9
HIV Nef LG13
HIV RT IL9
HIV Gag SL9
HIV RT TI9
HIV Mean
839W85

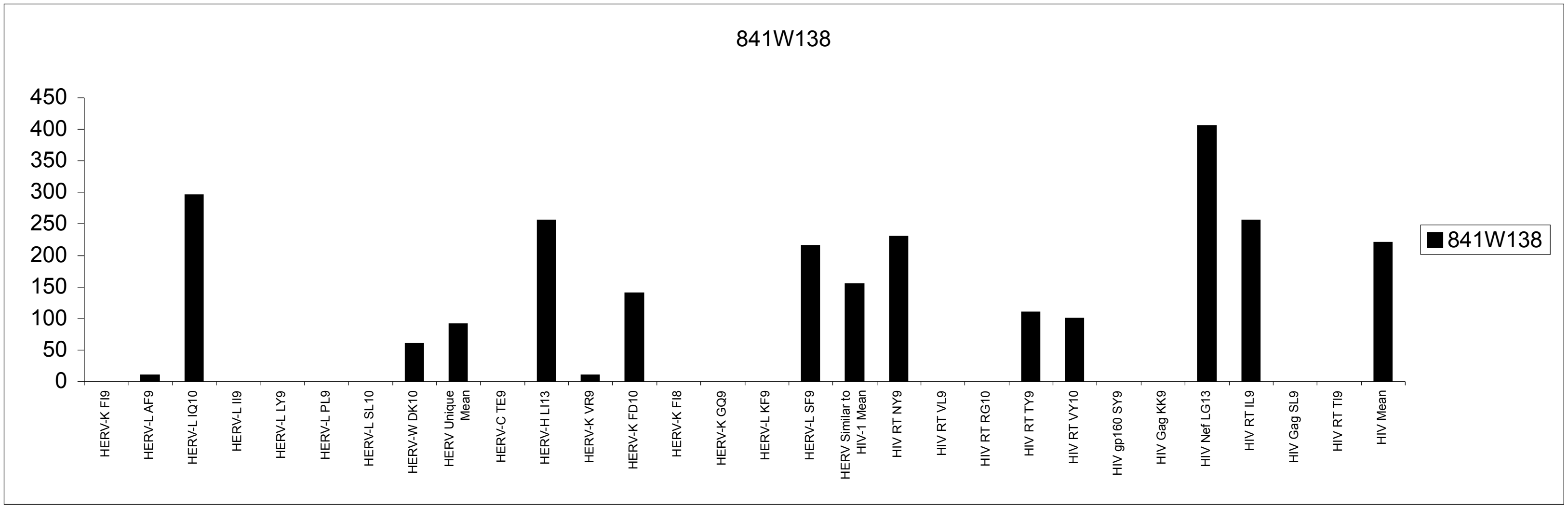

841W138
450
400
350
300
250
200
150
100
50
0
HERV-K FI9
HERV-L AF9
HERV-L IQ10
HERV-L II9
HERV-L LY9
HERV-L PL9
HERV-L SL10
HERV-W DK10
HERV Unique Mean
HERV-C TE9
HERV-H LI13
HERV-K VR9
HERV-K FD10
HERV-K FI8
HERV-K GQ9
HERV-L KF9
HERV-L SF9
HERV Similar to HIV-1 Mean
HIV RT NY9
HIV RT VL9
HIV RT RG10
HIV RT TY9
HIV RT VY10
HIV gp160 SY9
HIV Gag KK9
HIV Nef LG13
HIV RT IL9
HIV Gag SL9
HIV RT TI9
HIV Mean
841W138

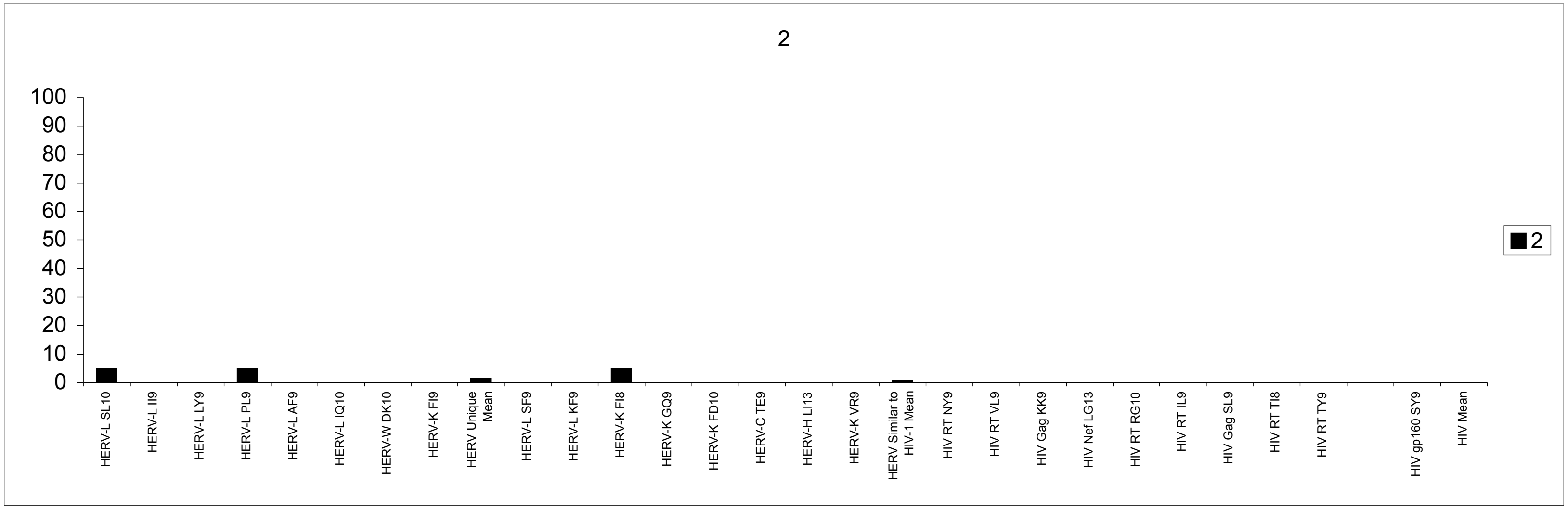
2
100
90
80
70
60
50
40
30
20
10
0
HERV-L SL10
HERV-L II9
HERV-L LY9
HERV-L PL9
HERV-L AF9
HERV-L IQ10
HERV-W DK10
HERV-K FI9
HERV Unique Mean
HERV-L SF9
HERV-L KF9
HERV-K FI8
HERV-K GQ9
HERV-K FD10
HERV-C TE9
HERV-H LI13
HERV-K VR9
HERV Similar to HIV-1 Mean
HIV RT NY9
HIV RT VL9
HIV Gag KK9
HIV Nef LG13
HIV RT RG10
HIV RT IL9
HIV Gag SL9
HIV RT TI8
HIV RT TY9
HIV gp160 SY9
HIV Mean
2

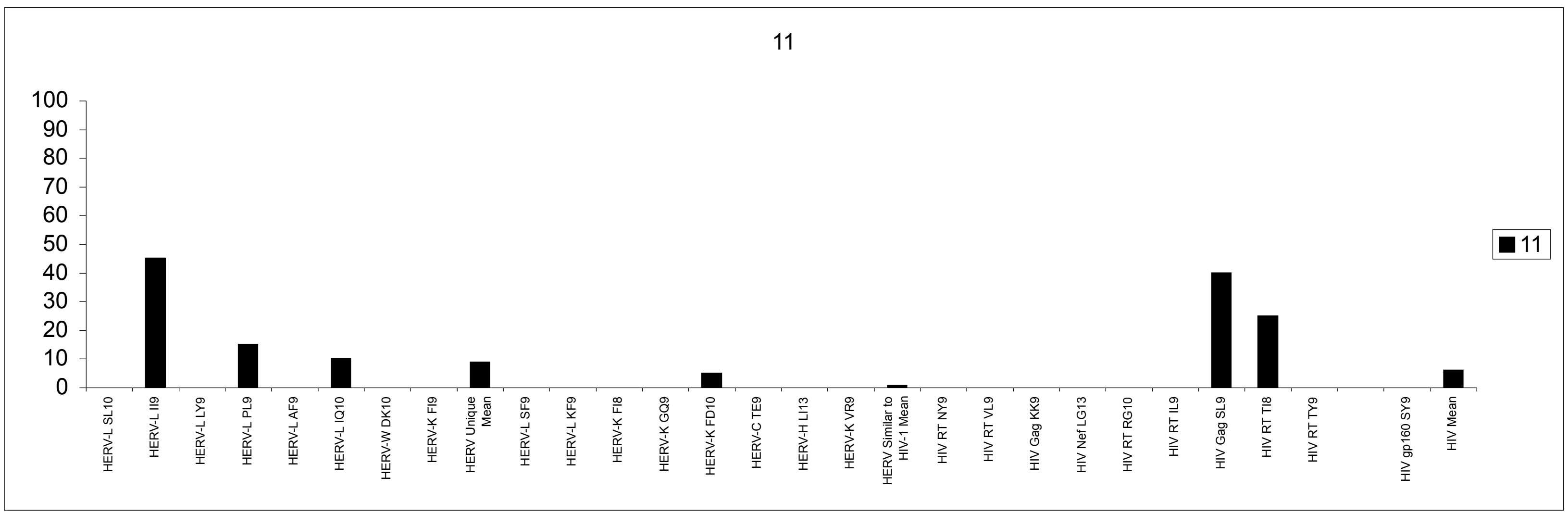
11
100
90
80
70
60
50
40
30
20
10
0
HERV-L SL10
HERV-L II9
HERV-L LY9
HERV-L PL9
HERV-L AF9
HERV-L IQ10
HERV-W DK10
HERV-K FI9
HERV Unique Mean
HERV-L SF9
HERV-L KF9
HERV-K FI8
HERV-K GQ9
HERV-K FD10
HERV-C TE9
HERV-H LI13
HERV-K VR9
HERV Similar to HIV-1 Mean
HIV RT NY9
HIV RT VL9
HIV Gag KK9
HIV Nef LG13
HIV RT RG10
HIV RT IL9
HIV Gag SL9
HIV RT TI8
HIV RT TY9
HIV gp160 SY9
HIV Mean
11

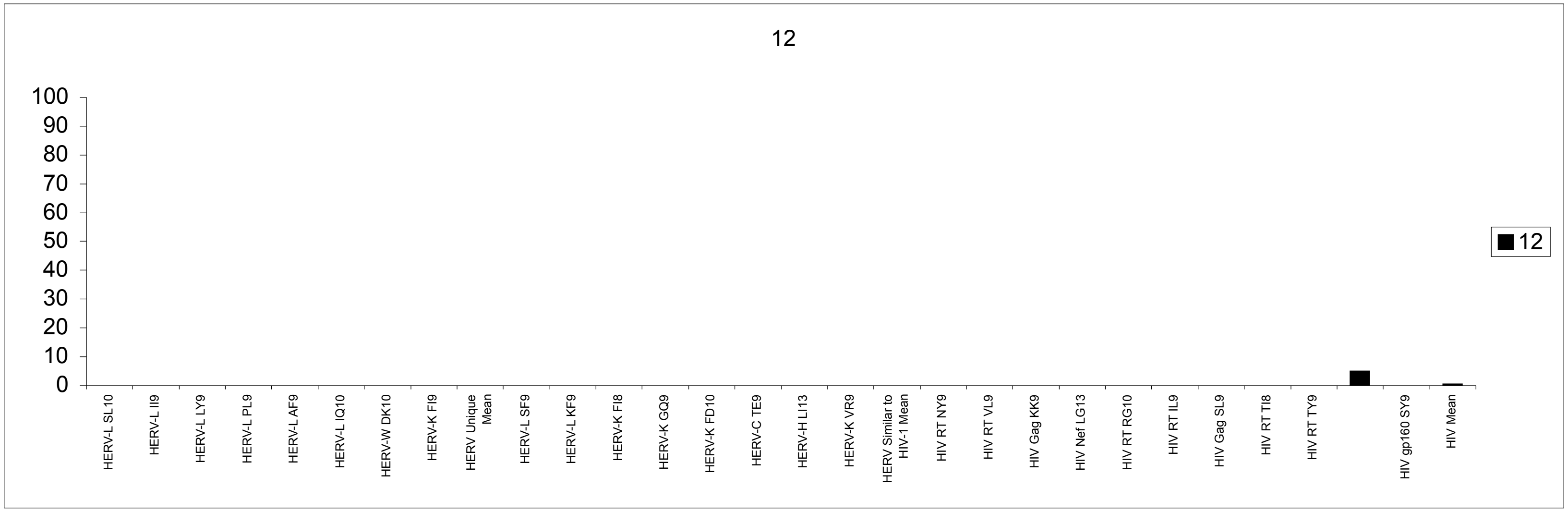
12
100
90
80
70
60
50
40
30
20
10
0
HERV-L SL10
HERV-L II9
HERV-L LY9
HERV-L PL9
HERV-L AF9
HERV-L IQ10
HERV-W DK10
HERV-K FI9
HERV Unique Mean
HERV-L SF9
HERV-L KF9
HERV-K FI8
HERV-K GQ9
HERV-K FD10
HERV-C TE9
HERV-H LI13
HERV-K VR9
HERV Similar to HIV-1 Mean
HIV RT NY9
HIV RT VL9
HIV Gag KK9
HIV Nef LG13
HIV RT RG10
HIV RT IL9
HIV Gag SL9
HIV RT TI8
HIV RT TY9
HIV gp160 SY9
HIV Mean
12

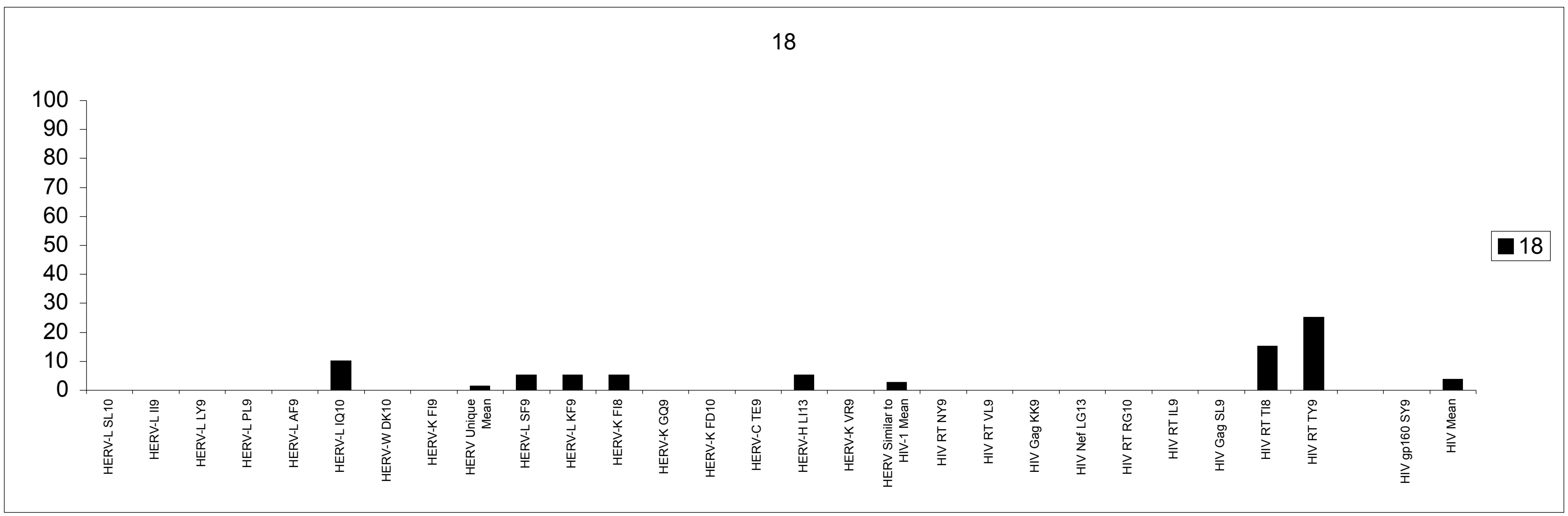
18
100
90
80
70
60
50
40
30
20
10
0
HERV-L SL10
HERV-L II9
HERV-L LY9
HERV-L PL9
HERV-L AF9
HERV-L IQ10
HERV-W DK10
HERV-K FI9
HERV Unique Mean
HERV-L SF9
HERV-L KF9
HERV-K FI8
HERV-K GQ9
HERV-K FD10
HERV-C TE9
HERV-H LI13
HERV-K VR9
HERV Similar to HIV-1 Mean
HIV RT NY9
HIV RT VL9
HIV Gag KK9
HIV Nef LG13
HIV RT RG10
HIV RT IL9
HIV Gag SL9
HIV RT TI8
HIV RT TY9
HIV gp160 SY9
HIV Mean
18

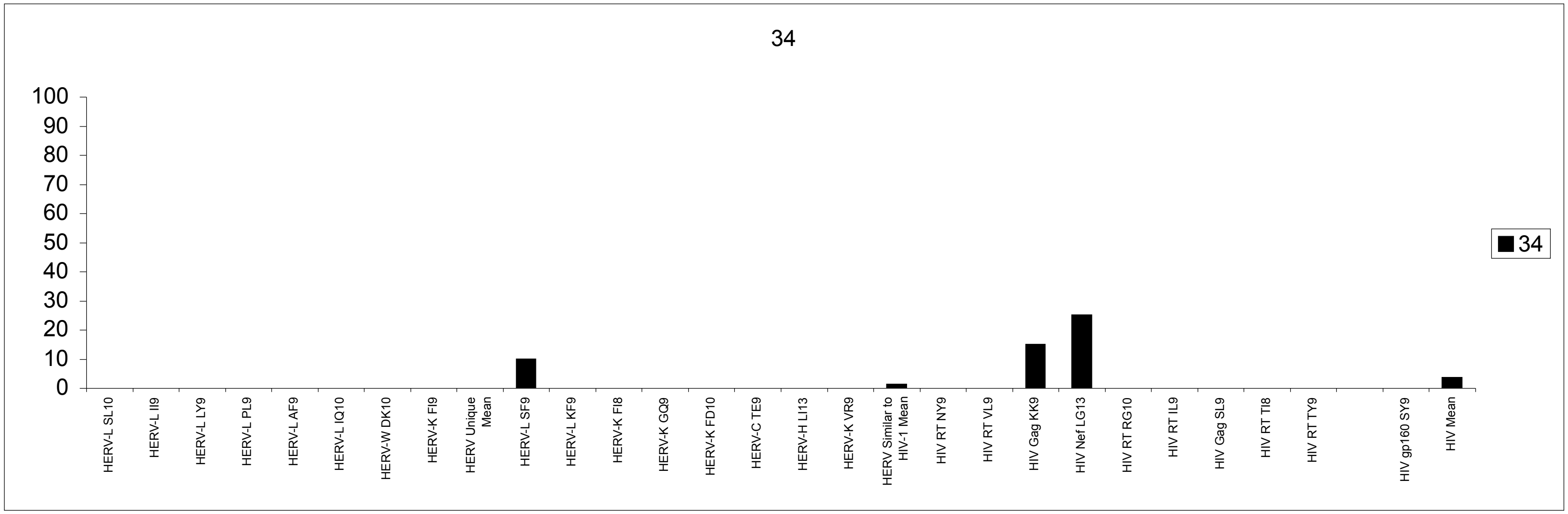
34
100
90
80
70
60
50
40
30
20
10
0
HERV-L SL10
HERV-L II9
HERV-L LY9
HERV-L PL9
HERV-L AF9
HERV-L IQ10
HERV-W DK10
HERV-K FI9
HERV Unique Mean
HERV-L SF9
HERV-L KF9
HERV-K FI8
HERV-K GQ9
HERV-K FD10
HERV-C TE9
HERV-H LI13
HERV-K VR9
HERV Similar to HIV-1 Mean
HIV RT NY9
HIV RT VL9
HIV Gag KK9
HIV Nef LG13
HIV RT RG10
HIV RT IL9
HIV Gag SL9
HIV RT TI8
HIV RT TY9
HIV gp160 SY9
HIV Mean
34

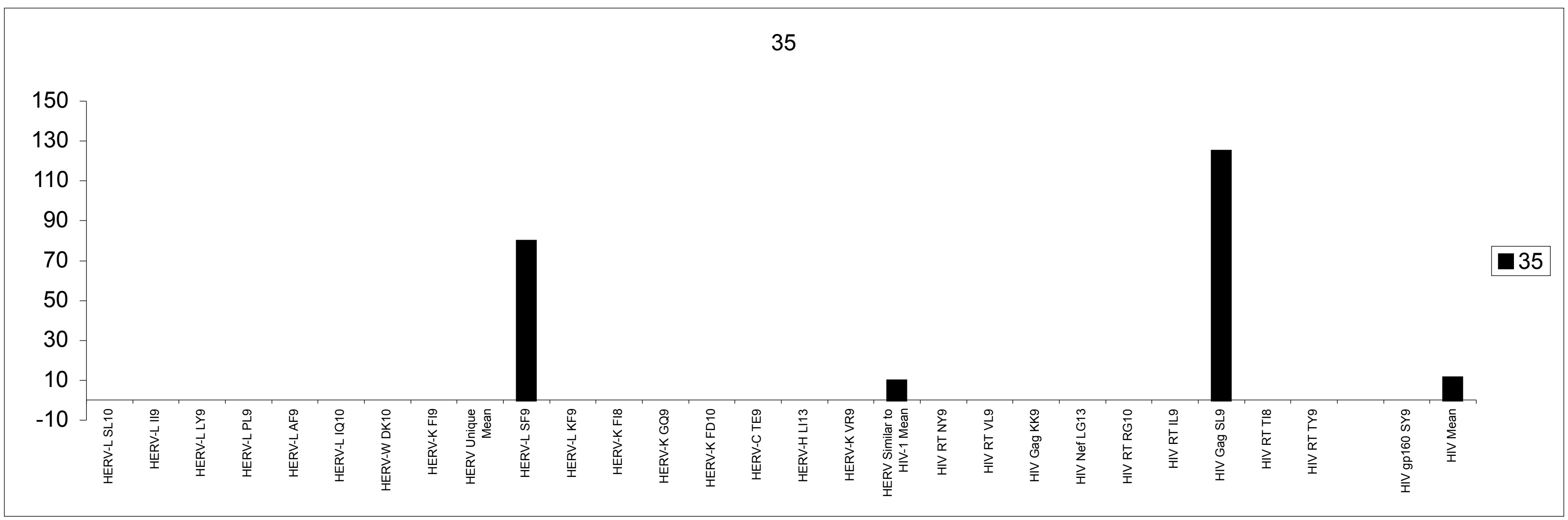
35
150
130
110
90
70
50
30
10
-10
HERV-L SL10
HERV-L II9
HERV-L LY9
HERV-L PL9
HERV-L AF9
HERV-L IQ10
HERV-W DK10
HERV-K FI9
HERV Unique Mean
HERV-L SF9
HERV-L KF9
HERV-K FI8
HERV-K GQ9
HERV-K FD10
HERV-C TE9
HERV-H LI13
HERV-K VR9
HERV Similar to HIV-1 Mean
HIV RT NY9
HIV RT VL9
HIV Gag KK9
HIV Nef LG13
HIV RT RG10
HIV RT IL9
HIV Gag SL9
HIV RT TI8
HIV RT TY9
HIV gp160 SY9
HIV Mean
35

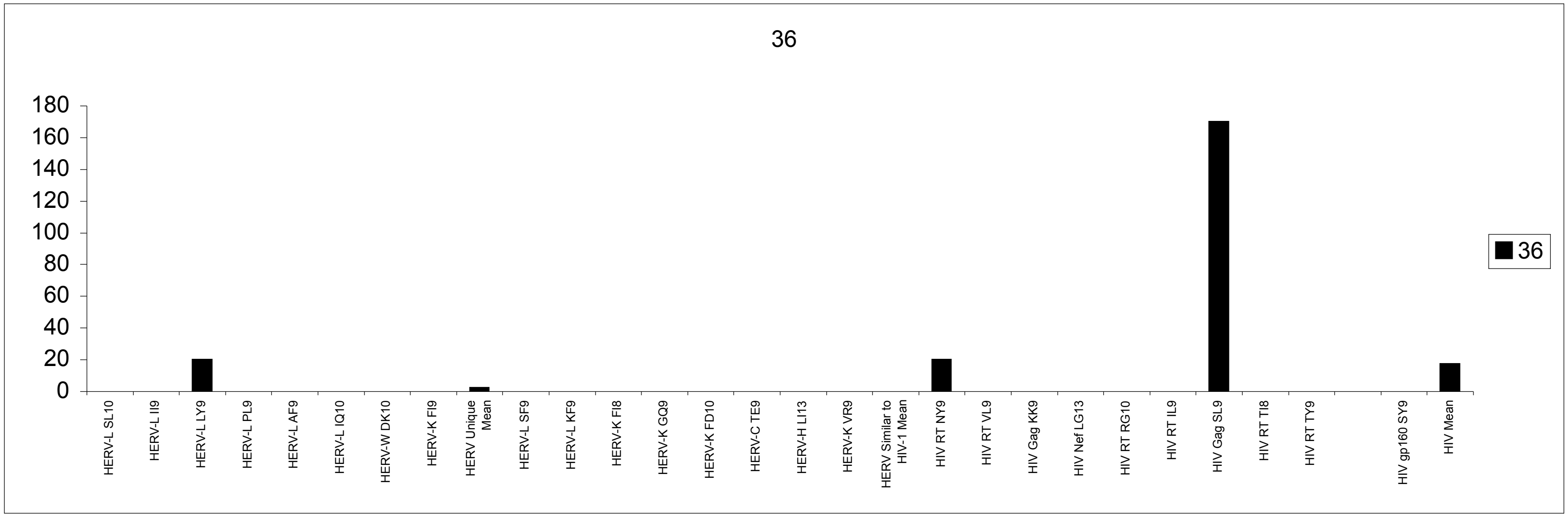
36
180
160
140
120
100
80
60
40
20
0
HERV-L SL10
HERV-L II9
HERV-L LY9
HERV-L PL9
HERV-L AF9
HERV-L IQ10
HERV-W DK10
HERV-K FI9
HERV Unique Mean
HERV-L SF9
HERV-L KF9
HERV-K FI8
HERV-K GQ9
HERV-K FD10
HERV-C TE9
HERV-H LI13
HERV-K VR9
HERV Similar to HIV-1 Mean
HIV RT NY9
HIV RT VL9
HIV Gag KK9
HIV Nef LG13
HIV RT RG10
HIV RT IL9
HIV Gag SL9
HIV RT TI8
HIV RT TY9
HIV gp160 SY9
HIV Mean
36

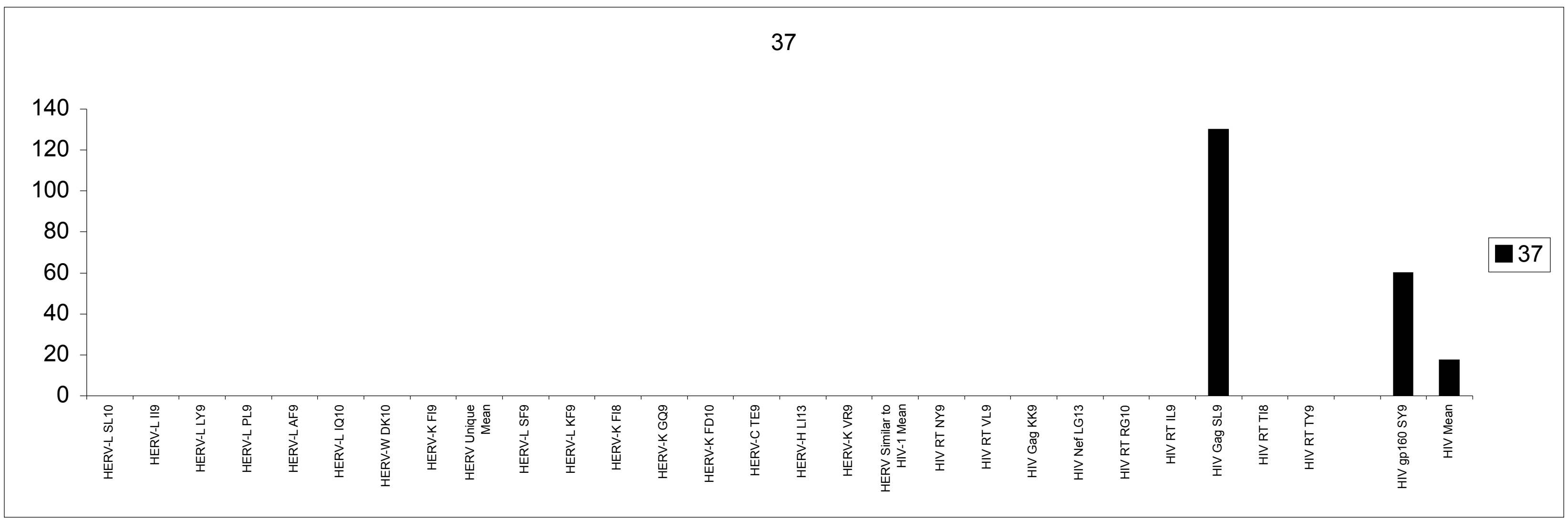
37
140
120
100
80
60
40
20
0
HERV-L SL10
HERV-L II9
HERV-L LY9
HERV-L PL9
HERV-L AF9
HERV-L IQ10
HERV-W DK10
HERV-K FI9
HERV Unique Mean
HERV-L SF9
HERV-L KF9
HERV-K FI8
HERV-K GQ9
HERV-K FD10
HERV-C TE9
HERV-H LI13
HERV-K VR9
HERV Similar to HIV-1 Mean
HIV RT NY9
HIV RT VL9
HIV Gag KK9
HIV Nef LG13
HIV RT RG10
HIV RT IL9
HIV Gag SL9
HIV RT TI8
HIV RT TY9
HIV gp160 SY9
HIV Mean
37

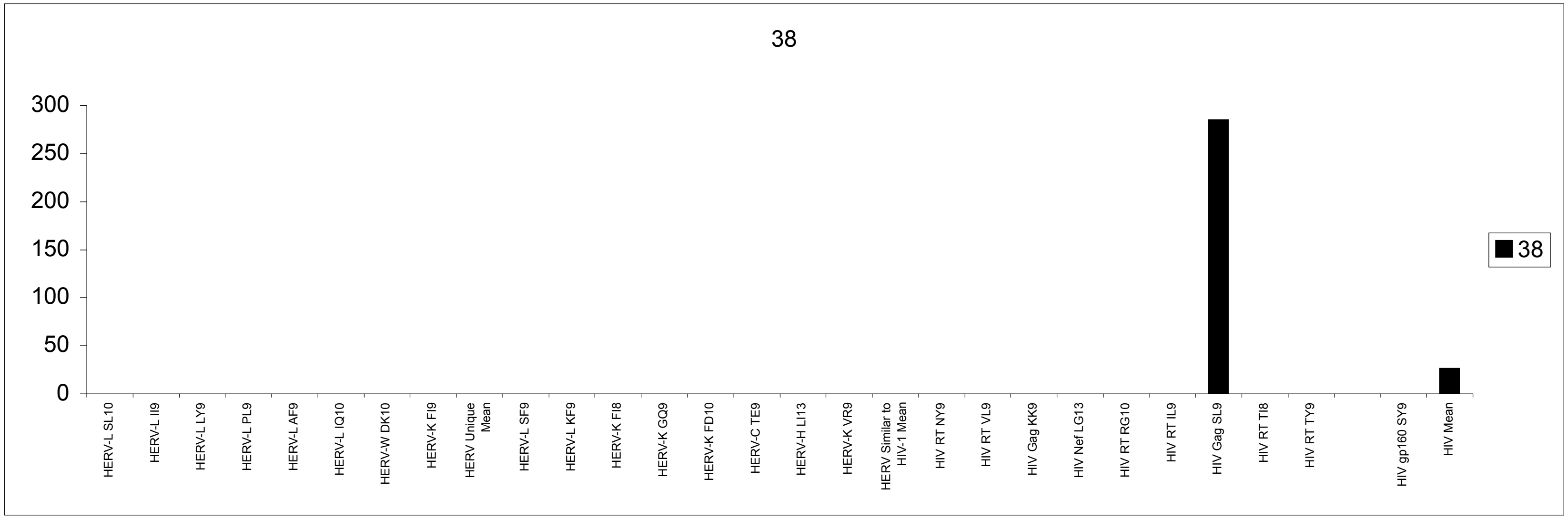
38
300
250
200
150
100
50
0
38
HERV-L SL10
HERV-L II9
HERV-L LY9
HERV-L PL9
HERV-L AF9
HERV-L IQ10
HERV-W DK10
HERV-K FI9
HERV Unique Mean
HERV-L SF9
HERV-L KF9
HERV-K FI8
HERV-K GQ9
HERV-K FD10
HERV-C TE9
HERV-H LI13
HERV-K VR9
HERV Similar to HIV-1 Mean
HIV RT NY9
HIV RT VL9
HIV Gag KK9
HIV Nef LG13
HIV RT RG10
HIV RT IL9
HIV Gag SL9
HIV RT TI8
HIV RT TY9
HIV gp160 SY9
HIV Mean

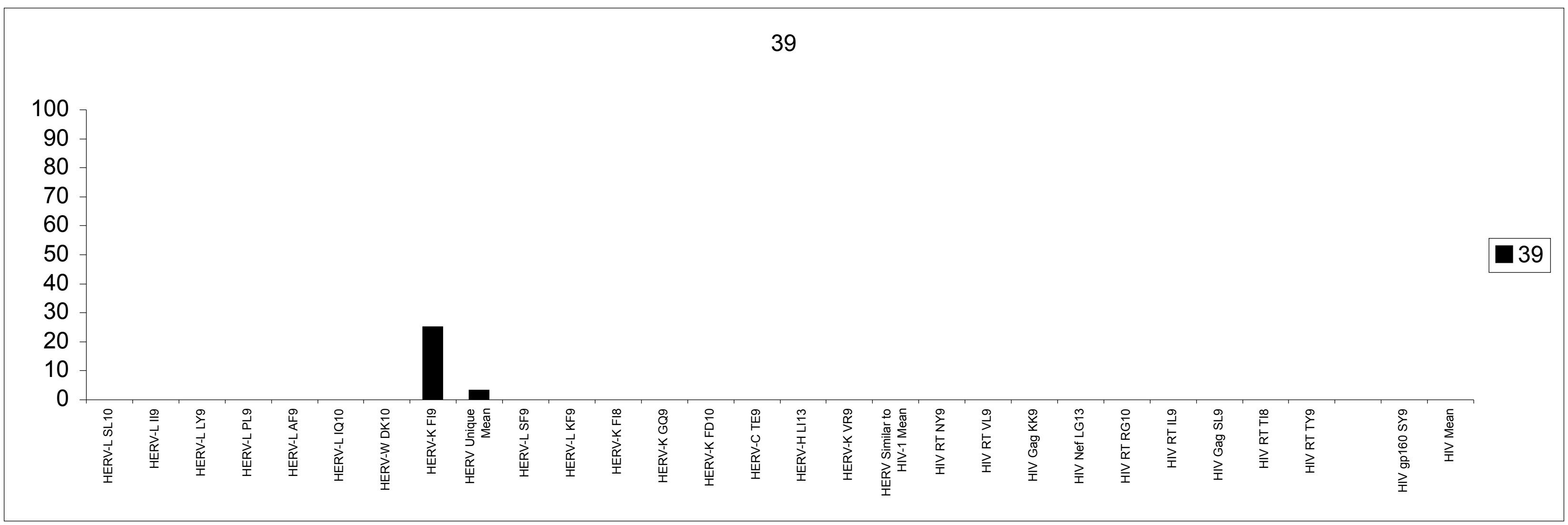
39
100
90
80
70
60
50
40
30
20
10
0
39
HERV-L SL10
HERV-L II9
HERV-L LY9
HERV-L PL9
HERV-L AF9
HERV-L IQ10
HERV-W DK10
HERV-K FI9
HERV Unique Mean
HERV-L SF9
HERV-L KF9
HERV-K FI8
HERV-K GQ9
HERV-K FD10
HERV-C TE9
HERV-H LI13
HERV-K VR9
HERV Similar to HIV-1 Mean
HIV RT NY9
HIV RT VL9
HIV Gag KK9
HIV Nef LG13
HIV RT RG10
HIV RT IL9
HIV Gag SL9
HIV RT TI8
HIV RT TY9
HIV gp160 SY9
HIV Mean

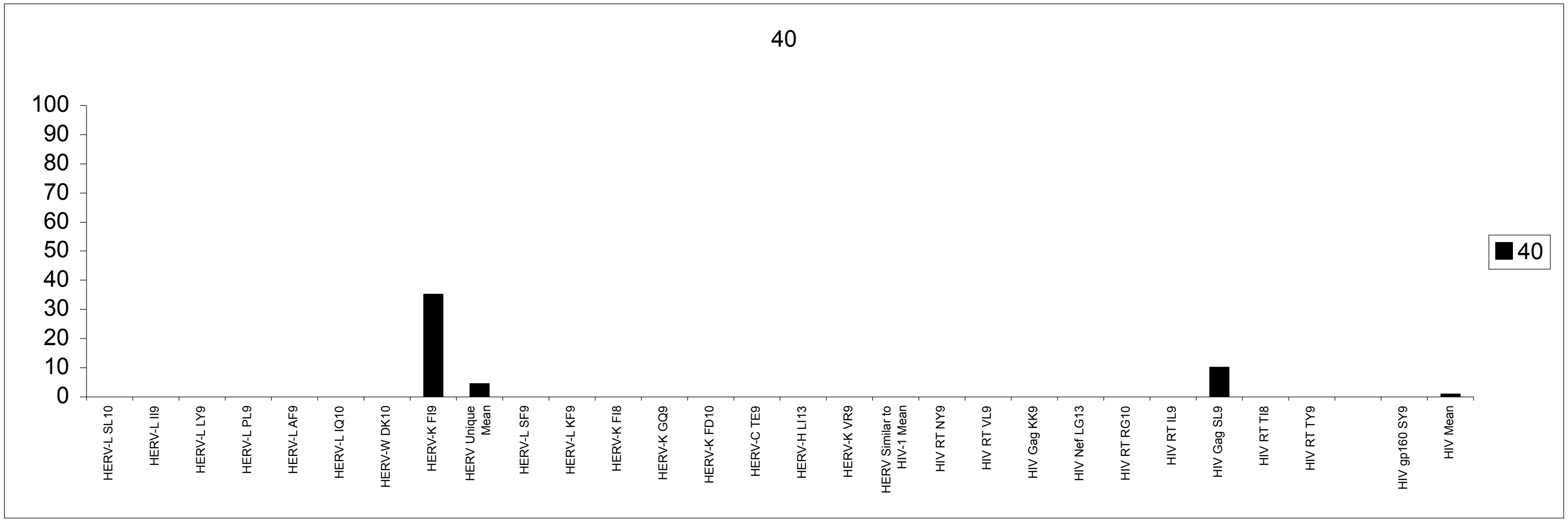

40
100
90
80
70
60
50
40
30
20
10
0
HERV-L SL10
HERV-L II9
HERV-L LY9
HERV-L PL9
HERV-L AF9
HERV-L IQ10
HERV-W DK10
HERV-K FI9
HERV Unique Mean
HERV-L SF9
HERV-L KF9
HERV-K FI8
HERV-K GQ9
HERV-K FD10
HERV-C TE9
HERV-H LI13
HERV-K VR9
HERV Similar to HIV-1 Mean
HIV RT NY9
HIV RT VL9
HIV Gag KK9
HIV Nef LG13
HIV RT RG10
HIV RT IL9
HIV Gag SL9
HIV RT TI8
HIV RT TY9
HIV gp160 SY9
HIV Mean
40

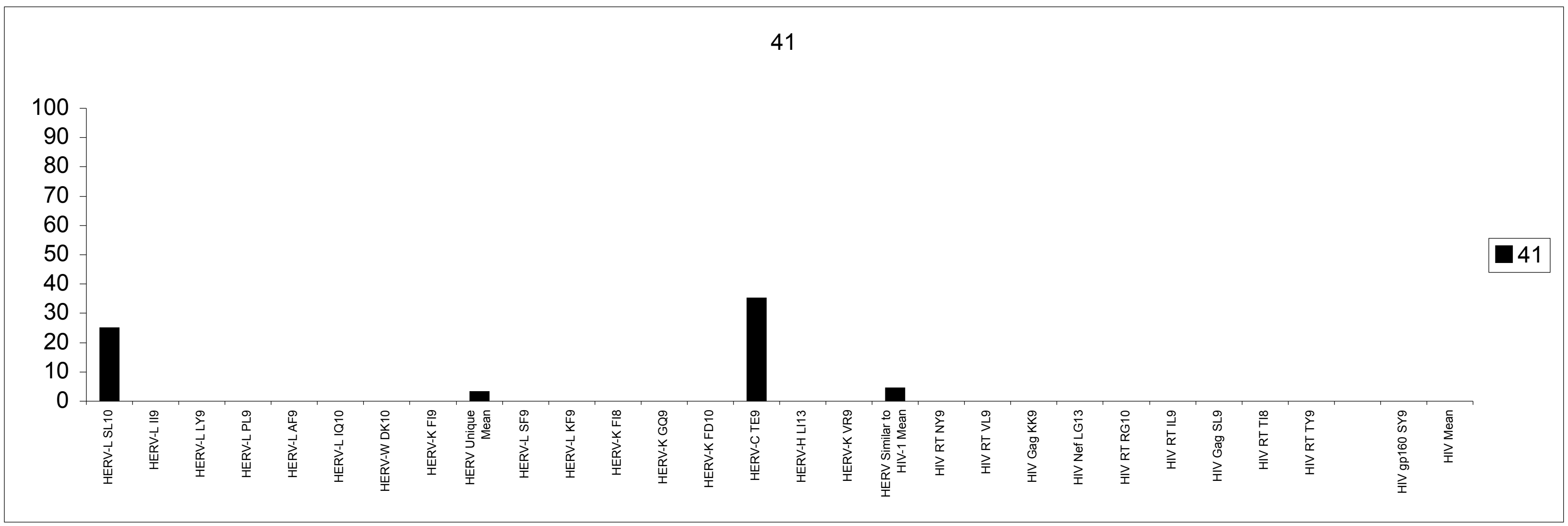

41
100
90
80
70
60
50
40
30
20
10
0
HERV-L SL10
HERV-L II9
HERV-L LY9
HERV-L PL9
HERV-L AF9
HERV-L IQ10
HERV-W DK10
HERV-K FI9
HERV Unique Mean
HERV-L SF9
HERV-L KF9
HERV-K FI8
HERV-K GQ9
HERV-K FD10
HERV-C TE9
HERV-H LI13
HERV-K VR9
HERV Similar to HIV-1 Mean
HIV RT NY9
HIV RT VL9
HIV Gag KK9
HIV Nef LG13
HIV RT RG10
HIV RT IL9
HIV Gag SL9
HIV RT TI8
HIV RT TY9
HIV gp160 SY9
HIV Mean
41

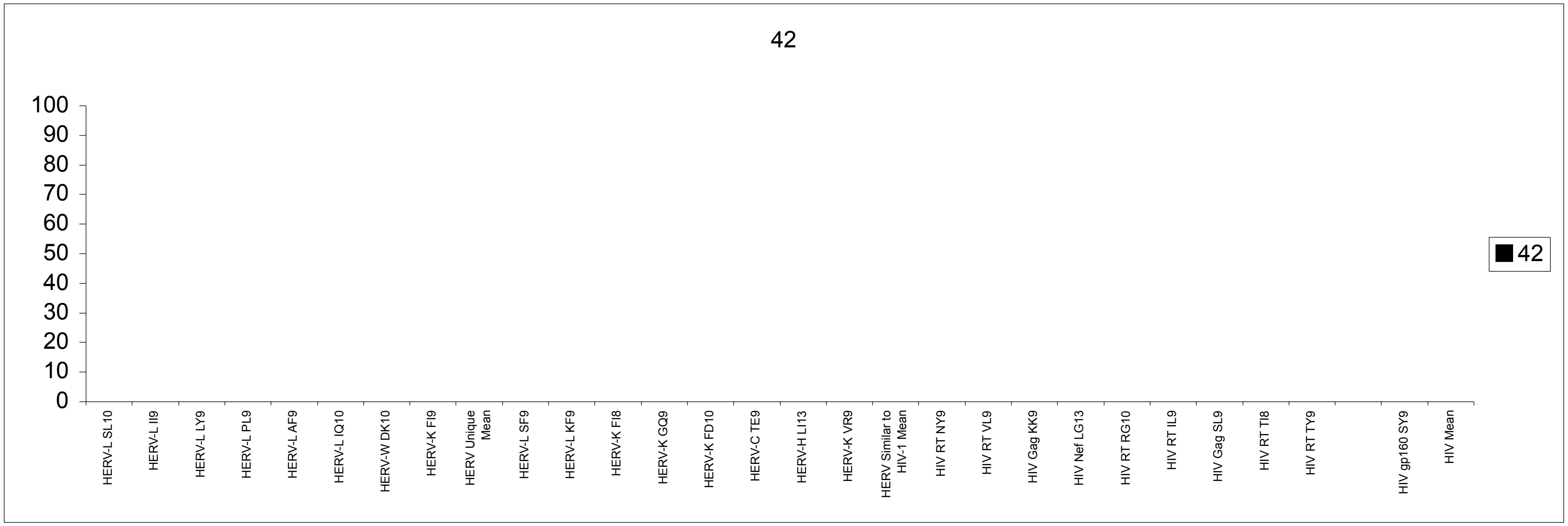

42
100
90
80
70
60
50
40
30
20
10
0
HERV-L SL10
HERV-L II9
HERV-L LY9
HERV-L PL9
HERV-L AF9
HERV-L IQ10
HERV-W DK10
HERV-K FI9
HERV Unique Mean
HERV-L SF9
HERV-L KF9
HERV-K FI8
HERV-K GQ9
HERV-K FD10
HERV-C TE9
HERV-H LI13
HERV-K VR9
HERV Similar to HIV-1 Mean
HIV RT NY9
HIV RT VL9
HIV Gag KK9
HIV Nef LG13
HIV RT RG10
HIV RT IL9
HIV Gag SL9
HIV RT TI8
HIV RT TY9
HIV gp160 SY9
HIV Mean
42

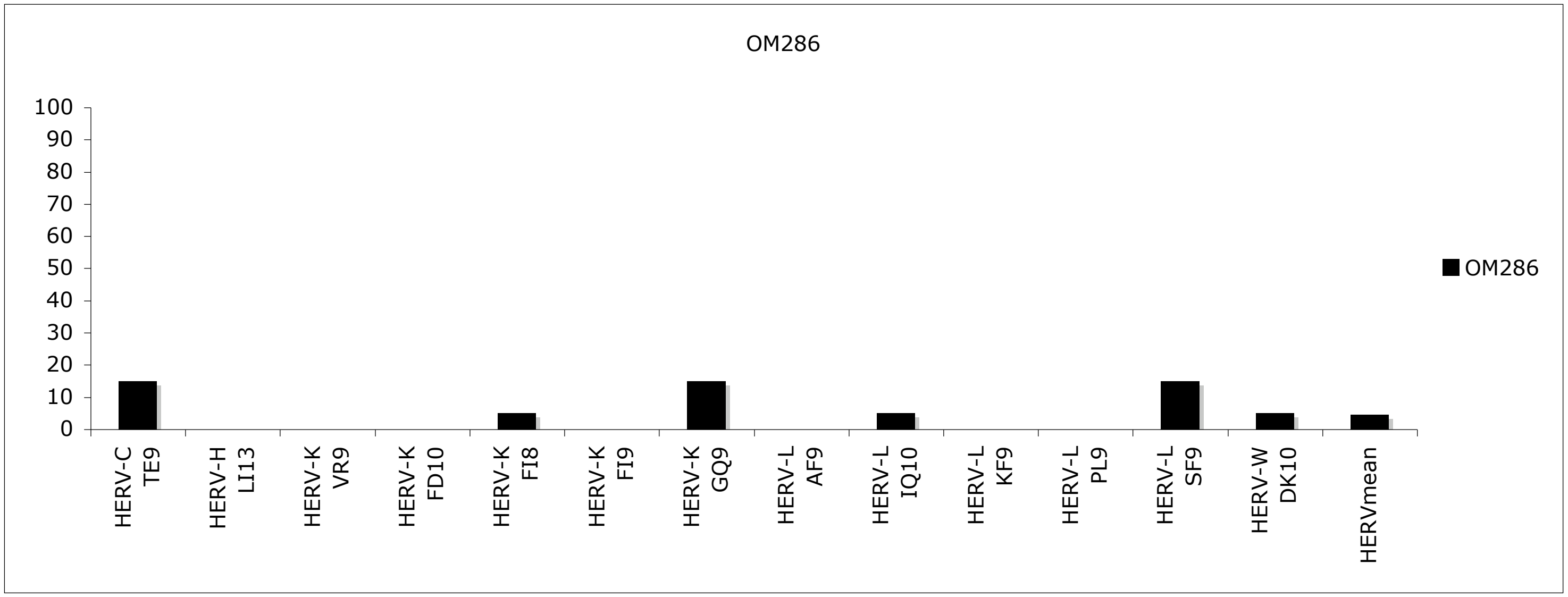
OM286
100
90
80
70
60
50
40
30
20
10
0
HERV-C TE9
HERV-H LI13
HERV-K VR9
HERV-K FD10
HERV-K FI8
HERV-K FI9
HERV-K GQ9
HERV-L AF9
HERV-L IQ10
HERV-L KF9
HERV-L PL9
HERV-L SF9
HERV-W DK10
HERVmean
OM286

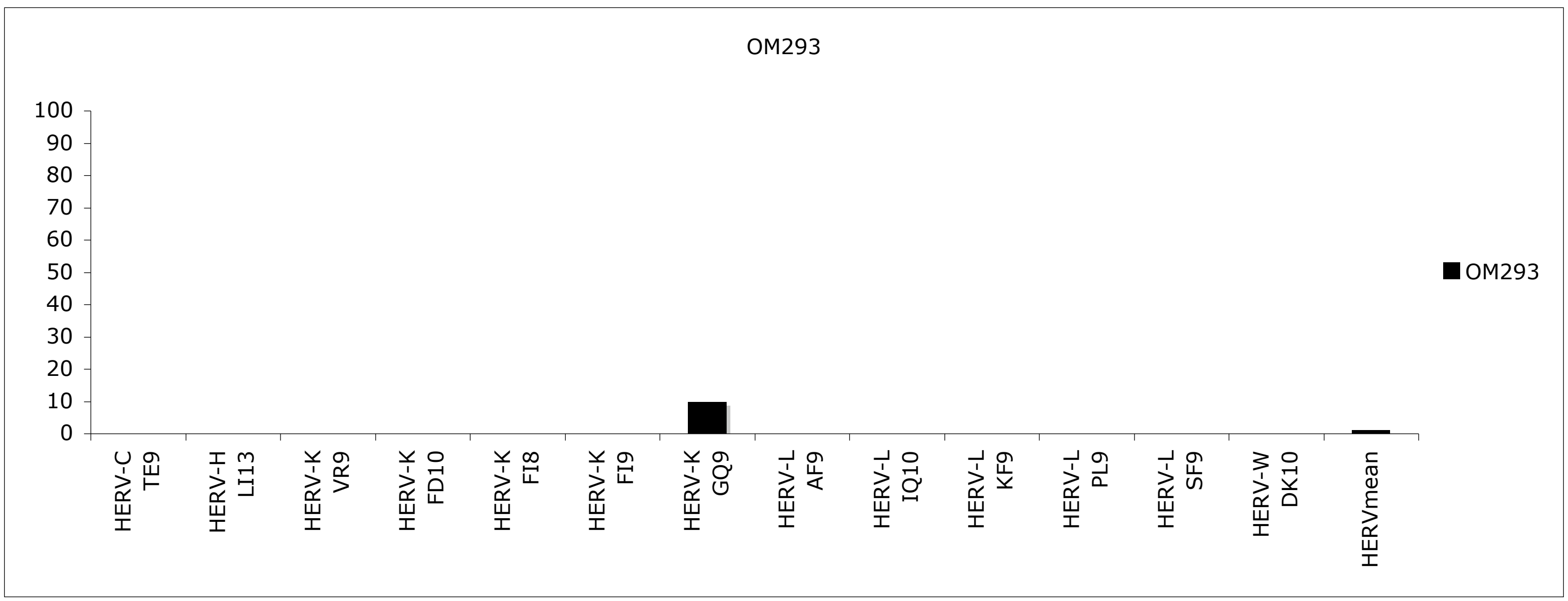
OM293
100
90
80
70
60
50
40
30
20
10
0
HERV-C TE9
HERV-H LI13
HERV-K VR9
HERV-K FD10
HERV-K FI8
HERV-K FI9
HERV-K GQ9
HERV-L AF9
HERV-L IQ10
HERV-L KF9
HERV-L PL9
HERV-L SF9
HERV-W DK10
HERVmean
OM293

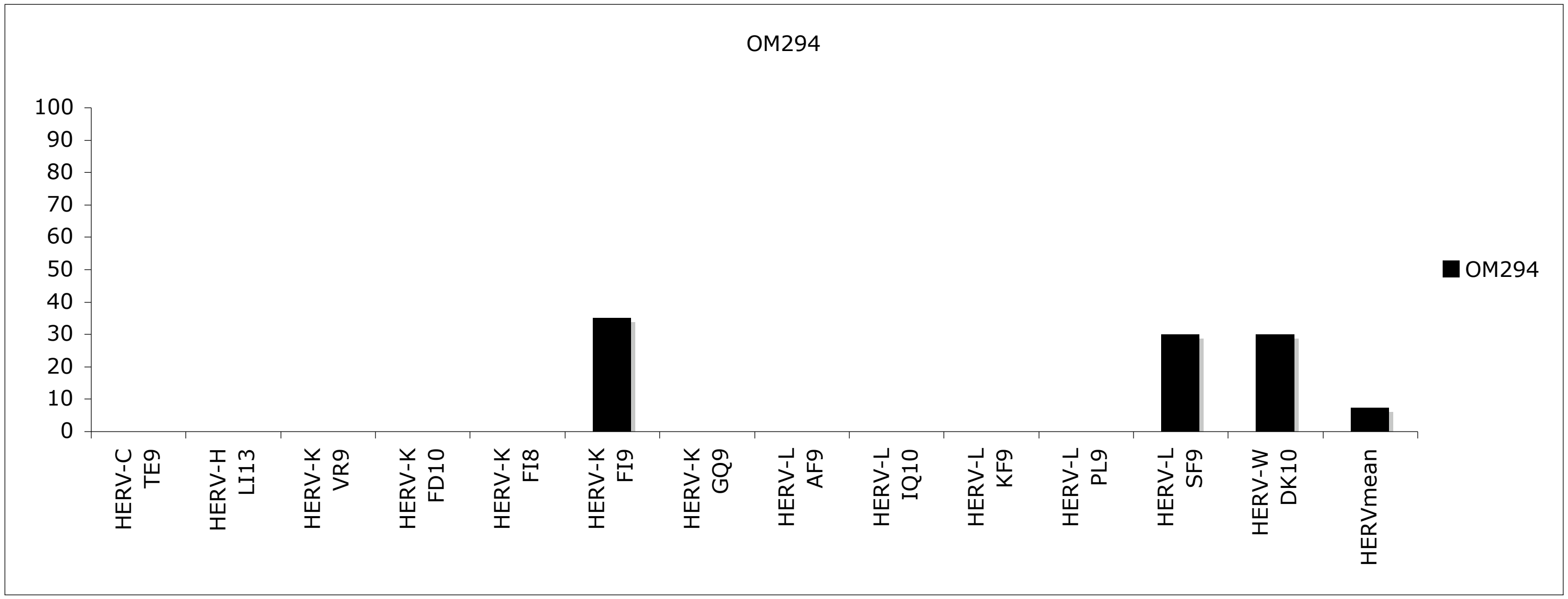
OM294
100
90
80
70
60
50
40
30
20
10
0
OM294
HERV-C TE9
HERV-H LI13
HERV-K VR9
HERV-K FD10
HERV-K FI8
HERV-K FI9
HERV-K GQ9
HERV-L AF9
HERV-L IQ10
HERV-L KF9
HERV-L PL9
HERV-L SF9
HERV-W DK10
HERVmean
