# Supplementary material for: T Cell Responses to Human Endogenous Retroviruses in HIV-1 Infection
Source: PLoS Pathog. 2007 Nov 9;3(11):e165. doi: 10.1371/journal.ppat.0030165 (PMC2065876; doi:10.1371/journal.ppat.0030165)
Supplement: Supplementary file 4 [file ppat.0030165.sd001.doc]

Supplemental Methods

**Identification of cytokines of interest for response comparisons between HIV-1, HERV and CMV-responding cells.** Cells from two HIV-1 positive subjects with T cell responses against HERV peptides identified in ELISPOT analysis were stimulated with the HERV-L derived peptide *HERV-L IQ10*. Production of Interferon-γ, TNF-α and IL-2 were measured in the cells following stimulation in parallel with cell surface CD107 expression [43]. The data from both HIV-1 positive subjects were analysed with the SPICE software to determine the cytokines or combinations which were associated with CD107 surface expression. Production of Interferon-γ and TNF-α together, thus associated with degranulation in response to the HERV peptides, was used for subsequent comparison of the phenotype of cells producing those same cytokines in response to other antigens.

**Determining the specificity of the primers used in the quantitative PCR assay for different HERV families.** To assess the level of specificity of the primers listed in Supplementary Table 2a, we performed direct sequencing on the PCR product obtained from the amplification. The sequences obtained from the PCR products along with their matches in the NCBI nr database [limited to organism: human (txid9606)] are shown in Supplemental Table 2b. Alignments of the env regions of selected HERV families (HERV-K, HERV-E, HERV-H, HERV-W) also indicated that primer binding was specific for the HERV-K family (data not shown).

**Expansion of CD8+ T cell effectors from HIV-1 negative study participants.** Effectors were expanded from HIV-1 individuals under the same conditions as the cells shown in Figure 7 (see Materials and Methods), and targets were an HLA-A2-expressing B cell line. As a positive control, a pool of CMV, EBV and flu peptides (CEF) was used as an antigen in the expansion of CD8+ effectors. Responses to the CEF pool are common, even in healthy study volunteers.
