# Supplementary material for: T Cell Responses to Human Endogenous Retroviruses in HIV-1 Infection
Source: PLoS Pathog. 2007 Nov 9;3(11):e165. doi: 10.1371/journal.ppat.0030165 (PMC2065876; doi:10.1371/journal.ppat.0030165)
Supplement: Supplementary file 5 [file ppat.0030165.st001.doc]

Supplementary Table 1. Additional peptide data including HERV subtypes with accession numbers and HIV HXB-2 protein location and HLA restrictions.

| Peptide Name | Amino acid sequence | HLA restriction | Start Position | Accession | Sequence Origin |
| --- | --- | --- | --- | --- | --- |
| **Unique HERV**  **Peptides** | | | | | |
| HERV-L II9 | ILVHYIDDI |  |  | 162563 | HERV-L |
| HERV-L LY9 | LQDIILVHY |  |  | 162563 | HERV-L |
| HERV-W DK10 | DSIEGQLILK |  |  | gi52000737 | HERV-W |
| HERV-L AF9 | AAIDLANAF |  |  | 162604 | HERV-L |
| HERV-L IQ10 | IPVHKAHKKQ | ^1^ |  | 162604 | HERV-L |
| HERV-L SL10 | SQGYINSPAL |  |  | 162563 | HERV-L |
| **HERV peptides**  **similar to HIV-1** | | | | | |
| HERV-K FD10 | FEGLVDTGAD^2^ |  |  | gi52001472 | HERV-K |
| HERV-L KF9 | KIRLPPGYF |  |  | 162613 | HERV-L |
| HERV-L SF9 | SSGLMLMEF^2^ |  |  | 162605 | HERV-L |
| HERV-K FI8 | FAFTIPAI^3^ |  |  | gi5802821 | HERV-K |
| HERV-H LI13 | LDLLTAEKGGLCI |  |  | gi44887889 | HERV-H |
| HERV-K VR9 | VPLTKEQVR |  |  | gi5802821 | HERV-K |
| HERV-L PL9 | PMVSTPATL |  |  | 162568 | HERV-L |
| HERV-K TE9 | TLEPIPPGE |  |  | gi75766508 | HERV-K |
| HERV-K FI9 | FLQFKTWWI^2^ |  |  | Ref. [40] | HERV-K |
| HERV-K GQ9 | GIPYNSQGQ^2^ |  |  | gi67782351 | HERV-K |
| **HIV-1 peptides** | | | | | |
| HIV Gag KK9 | KIRLRPGGK | A3/B27 | Gag p17: 18 |  | HXB-2 |
| HIV RT TI9 | TAFTIPSI^3^ | B51 | RT p51: 128 |  | HXB-2 |
| HIV RT VL9 | VIYQYMDDL | A2 | RT p51: 179 |  | HXB2 |
| HIV Nef LG13 | LSHFLKEKGGLEG | Partial A3/A11 | Nef: 87 |  | HXB-2 |
| HIV RT IL9 | IPLTEEAEL | B3501^4^ | RT p51: 293 |  | HXB-2 |
| HIV Gag SL9 | SLYNTVATL | A2 | Gag p17: 77 |  | HXB-2 |
| HIV gp160 SY9 | SFEPIPIHY | A29 | gp160: 209 |  | HXB-2 |
| HIV RT NY9 | NPDIVIYQY | B3501^4^ | RT p51: 175 |  | HXB-2 |
| HIV RT TY9 | TVLDVGDAY | B35 | RT p51: 107 |  | HXB2 |
| HIV RT VY10 | VPLDEDFRKY | B3501^4^ | RT p51: 118 |  | HXB2 |
| HIV RT RG10 | RYQYNVLPQG | B51 | RT p51: 143 |  | HXB-2 |

Table Footnotes:

^1^ Likely presented on HLA-B35PX [1].

^2^ Possible conservation of HLA anchor residues with the most closely matching HIV-1 peptide, but that HIV-1 peptide is not a known epitope.

^3^ Conservation between HIV-1 and HERV peptide in HLA anchor residues [2].

^4^ Presented by HLA-B35PY(3501) [1,3]

**References**

1. Gao X, Nelson GW, Karacki P, Martin MP, Phair J, et al. (2001) Effect of a single amino acid change in MHC class I molecules on the rate of progression to AIDS. N Engl J Med 344: 1668-1675.

2. Maenaka K, Maenaka T, Tomiyama H, Takiguchi M, Stuart DI, et al. (2000) Nonstandard peptide binding revealed by crystal structures of HLA-B*5101 complexed with HIV immunodominant epitopes. J Immunol 165: 3260-3267.

3. Tomiyama H, Miwa K, Shiga H, Moore YI, Oka S, et al. (1997) Evidence of presentation of multiple HIV-1 cytotoxic T lymphocyte epitopes by HLA-B*3501 molecules that are associated with the accelerated progression of AIDS. J Immunol 158: 5026-5034.
