# Supplementary material for: T Cell Responses to Human Endogenous Retroviruses in HIV-1 Infection
Source: PLoS Pathog. 2007 Nov 9;3(11):e165. doi: 10.1371/journal.ppat.0030165 (PMC2065876; doi:10.1371/journal.ppat.0030165)
Supplement: Supplementary file 6 [file ppat.0030165.st002.doc]

Supplementary Table 2a. Primer sequences for HERV amplification.

| Target | Forward primer | T_m_ (°C) | Reverse Primer | T_m_ (°C) |
| --- | --- | --- | --- | --- |
| HERV-K Env | CTAGGGAGAGCACCAGGATG | 60 | GGGGCAAGTTTTCCCTTTAG | 60 |
| B-actin | TCCCTTGCCATCCTAAAAGCCACC | 67 | CTGGGCCATTCTCCTTAGAGAGAAG | 66 |

Supplementary Table 2b. Sequences obtained from HERV-K Env amplification.

| Sequencing Primer | Sequence | Best BLAST hit (nr database) | Score | E-value |
| --- | --- | --- | --- | --- |
| HERV-K Env Fwd | TTAATGCCTGCAGTCCAAAATTGGTTGGTAGAAGTACCTACTGTCAGTCCCATCAGTAGATTCACTTATCACATGGTAAGCGGGATGTCACTCAGGCCACGGGTAAATTATTTACAAGACTTTTCTTATCAAAGATCATTAAAATTTAGACCTAAAGGGAAAACTTGCCCCAAA | Homo sapiens endogenous virus Human endogenous retrovirus K HERV-K50D, complete sequence  gi\|73697544\|gb\|DQ112152.1\|[73697544] | 315 | 1e-83 |
| HERV-K Env Rev | GTCTAATTTTAATGATCTTTGATAAGAAAAGTCTTGTAAATAATTTACCCGTGGCCTGAGTGACATCCCGCTTACCATGTGATAAGTGAATCTACTGATGGGACTGACAGTAGGTACTTCTACCAACCAATTTTGGACTGCAGGCATTAAACATCCTGGTGCTCTCCCTAGAAAA | Homo sapiens endogenous virus Human endogenous retrovirus K HERV-K50D, complete sequence  gi\|73697544\|gb\|DQ112152.1\|[73697544] | 311 | 1e-82 |
